# Prevalence and risk factors of adverse birth outcomes in Bangladesh: Insight from a nationwide survey

**DOI:** 10.1371/journal.pone.0351676

**Published:** 2026-06-17

**Authors:** Ahadul Hassan Bhuiyan Konok, Md. Shahadoth Hossain, Md. Hafizul Islam

**Affiliations:** 1 Institute of Nutrition and Food Science, University of Dhaka, Dhaka, Bangladesh; 2 Department of Nutrition and Food Engineering, Daffodil International University, Dhaka, Bangladesh; 3 Center for Non-communicable Diseases and Nutrition, BRAC James P Grant School of Public Health, BRAC University, Dhaka, Bangladesh; Center for Evidence–Based Medicine and Clinical Research, BANGLADESH

## Abstract

**Background:**

Adverse birth outcomes are significant public health concern in Bangladesh and can severely affect the health and wellbeing of children in later life. This study aimed to assess the prevalence of adverse birth outcomes (i.e., stillbirth, preterm birth, low birth weight (LBW), neonatal death) and identify the associated factors among the Bangladeshi population.

**Methods:**

The study utilized nationally representative data from the Bangladesh Demographic and Health Survey (BDHS) to analyze index pregnancy outcomes for 10,254 ever-married women. The prevalence and associated factors of both composite and individual adverse outcomes were determined using appropriate statistical procedures.

**Results:**

The findings indicate that 14.3% of pregnancies resulted in LBW, while 8.9% resulted in preterm birth, followed by stillbirths (1.3%) and neonatal deaths (1.3%). Overall, 14.2% of births were associated with at least one adverse outcome. Household wealth index, place of delivery, twin births, maternal desire, and regional factors were found to be associated with LBW. Factors affecting preterm birth included cesarean section delivery, wealth index, twin births and maternal desire to have children. Regarding stillbirths, associations were found with the cesarean section delivery, and twin births. For neonatal death, factors associated included cesarean section delivery, twin birth, and maternal desire. Lastly, the composite score of adverse birth outcomes was associated with wealth index, history of terminated pregnancies, place of delivery, decision-making autonomy, region, twin births, and maternal desire.

**Conclusion:**

One in seven births in Bangladesh involved at least one adverse outcome, with LBW being the most prevalent. Socioeconomic disadvantage and limited women’s decision-making autonomy were consistently associated with adverse outcomes. Policies should prioritize equity-oriented maternal care, strengthen women’s autonomy, and ensure appropriate use of obstetric interventions.

## Introduction

Every woman aspires to have a joyful pregnancy; however, it may sometimes result in adverse outcomes, such as fetal loss during early or late gestation, premature delivery, or the birth of a baby with a lower-than-expected weight [1]. Adverse birth outcomes, such as stillbirth (fetal death occurring after 28 weeks of pregnancy, either in the womb or during labor), preterm birth (delivery before 37 weeks of gestation), low birth weight (LBW) (less than 2.5 kg at birth), and neonatal death (infant mortality within the first 28 days of life), are significant public health issues of global concern [2,3]. These are common issues in low- and middle-income countries (LMICs), particularly in resource-limited settings where access to and utilization of healthcare services are inadequate, leading to poorer childhood outcomes and serving as a primary cause of child mortality and morbidity [4,5].

In 2019, an estimated 2 million stillbirths were reported globally, corresponding to a stillbirth rate of approximately 13.9 per 1,000 total live births [6]. Importantly, approximately 84% of all stillbirths occur in LMICs [7]. The stillbirth rate in South Asia was significantly higher than the global average, at 18.2 per 1,000 total live births, with Bangladesh reporting an alarming rate of 24.3 per 1,000 total live births [6]. Globally, an estimated 15 million babies are born preterm each year [8], with 13.3% of these births occurring in South Asia [9]. Preterm birth is a leading cause of child mortality, contributing to the deaths of approximately one million children under the age of five annually. Literature shows that 17.2% of newborn deaths in Bangladesh are due to complications of preterm birth [10]. The global prevalence of LBW was 14.7%, while it was much higher in Southern Asia, where around 8.8 million babies were born with LBW [11]. Recent demographic and health survey shows that the prevalence of LBW in Bangladesh is 14.4% [12]. According to the global report, about 2.9 million newborns die within the first 28 days of their lives [7]. The situation is not good in Bangladesh; 20 out of every 1000 children die before the completion of the first month of their life [12].

Adverse birth outcomes significantly affect the health and well-being of individuals from infancy through adulthood [13]. Babies who experience adverse birth outcomes are at a higher risk of mortality and face a range of health and developmental challenges [3]. Preterm birth, one of the leading causes of infant mortality, is strongly linked to long-term consequences such as poor health and growth, mental and intellectual disabilities, early onset of chronic diseases like cardiovascular disease or diabetes, and physical or neurological issues, which may result in lifelong disabilities [3,14,15]. Similarly, LBW, which can also result from preterm birth, often leads to both physical and cognitive growth delays. Additionally, it can result in severe health problems, from immediately after birth to later life, and increases the risk of fetal and neonatal mortality [7,13]. Due to these turnovers, adverse birth outcomes impose a substantial economic burden on both families and the nation [16].

Numerous studies have been conducted in LMICs, including those in South Asia, to identify the factors associated with various adverse birth outcomes. The existing literature highlights several potential factors, including place of residence [2,5], maternal education [2,7], maternal age at childbirth [2,5], wealth index [2,7,13], birth order [2,7,9], parity [2], birth interval [2,3,13], unwanted pregnancies [3,13], place of delivery [7,13], geographic region [2], history of miscarriage and/or abortion [2,3,7], decision-making autonomy [7,9], maternal desire for pregnancy [2], media exposure [7,13], all of which may serve as predictors of adverse birth outcomes.

Bangladesh has significantly reduced maternal and child mortality in recent decades. However, LBW and child undernutrition rates (stunting-24%, wasting-11%, underweight-22%, etc.) among children under five are still a concern [12]. Evidence suggests that adverse birth outcomes could contribute to persistent child undernutrition, making their reduction a critical priority at both national and global levels [17,18]. Understanding the causes and contributing factors of these adverse birth outcomes could guide policy decisions to significantly reduce adverse birth outcomes and, eventually, child mortality and undernutrition in Bangladesh. While some studies have partially examined adverse birth outcomes and their determinants in Bangladesh [2,9,15,19], there is a lack of recent data on their distribution and determinants. Moreover, comprehensive studies on stillbirth, preterm birth, LBW and neonatal death simultaneously using a single nationally representative survey are rare. Additionally, in Bangladesh, the overall burden of composite adverse birth outcomes and their contributing factors remains unexplored. Therefore, this study aimed to assess the prevalence of adverse birth outcomes (i.e., stillbirth, preterm birth, LBW, neonatal death) and identify the associated factors in the Bangladeshi population.

## Methodology

### Study design and source of data

The study conducted a secondary analysis of nationally representative data to investigate adverse birth outcomes and their associated sociodemographic factors in Bangladesh. Data were obtained from the 2022 Bangladesh Demographic and Health Survey (BDHS) [20]. The sampling process employed a two-stage stratified design to ensure national representativeness. In the first stage, 675 enumeration areas (EAs) were selected (237 from urban areas and 438 from rural areas) using probability proportional to the size of each EA. In the second stage, a systematic sample of approximately 45 households per EA was drawn to provide statistically reliable estimates across all eight divisions and for rural and urban areas separately. From the identified households, 30 were randomly chosen for interviews. Ultimately, data were collected from 674 clusters (EAs), encompassing all eight divisions of Bangladesh. Further details on the sampling methodology are available in the BDHS-2022 report [12].

### Study sample

**Fig 1:**
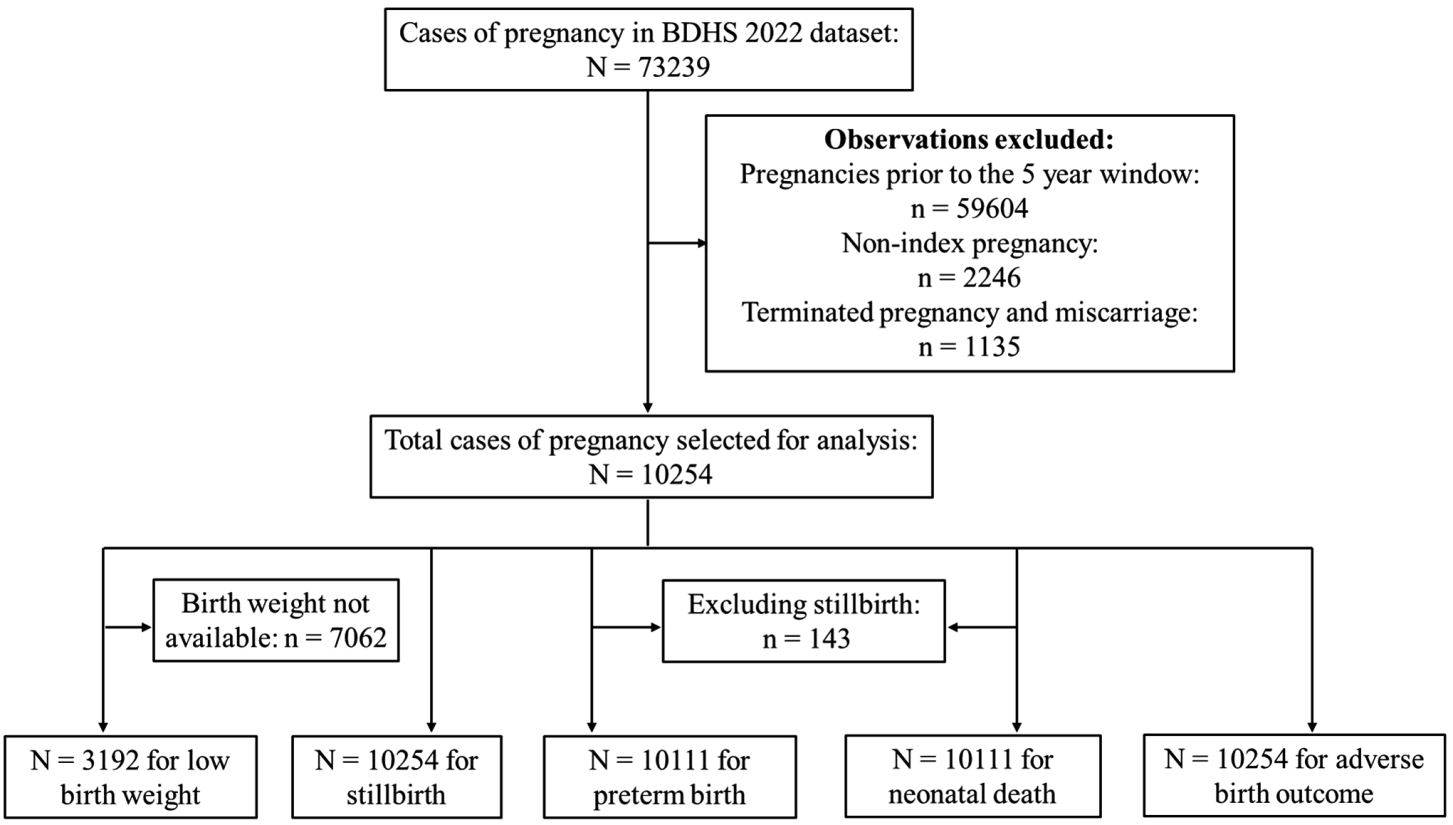
Sample selection procedure for the outcome variables.

The BDHS included ever-married women aged 15–49 years. The primary inclusion criteria of the present study were women who gave at least one birth within five years preceding the survey. In the pregnancy record file (GR) a total of 73,239 pregnancy cases were available. From this, women with pregnancy before 5 years of the survey date (n = 59,604), non-index pregnancy (birth that are not most recent) (n = 2,246), and terminated pregnancy/miscarriage (n = 1,135) were excluded (Fig 1). Only the index pregnancy, defined as the most recent birth, was included to minimize recall bias. Terminated pregnancies and miscarriages were excluded because they occurred prior to the outcome period, and the study outcomes could not occur for these cases. Thus, the total cases of pregnancy selected for analysis were 10,254 (weighted observations 10,300). Respondents with missing data in any of the considered outcomes were also excluded from the analysis of that outcome servey. Finally, we obtained 3,192 cases for low birth weight, 10,254 for stillbirth, and 10,111 for preterm birth and neonatal death. The sample selection procedure for the outcome variables is given in Fig 1, and the proportion of missing data for each outcome and predictor variable is reported in the Supplementary Table 4 in S1 File.

### Outcome variables

In the present study, adverse birth outcomes were the outcome variables that included stillbirths (fetal deaths in pregnancies lasting seven or more months), neonatal death (deaths at age 0–28 days), preterm birth (birth before 37 weeks of gestation), and low birth weight (babies born with weight < 2500 gm). A composite variable (adverse birth outcome) was obtained from the above four outcomes, which is similar to the existing literature [5,7]. When a pregnancy was found having at least one of the above four outcomes, it was considered to have an adverse birth outcome. This composite outcome was intended to capture overall perinatal vulnerability across the late pregnancy and early neonatal period rather than to represent a single etiological process. Detailed operational definitions of outcome variables can be found in the Supplementary Table 1 in S1 File.

### Predictor variables

We considered a range of covariates to adjust the effects of exposure variables on outcome variables. We first conducted a comprehensive literature search using several databases. Relevant studies conducted in LMICs, particularly in Asian countries, were summarized. Factors found to have a significant association with adverse outcomes were listed. The availability of the listed variables was then checked with the BDHS-2022 dataset, and available variables were sorted out. The multicollinearity of the available variables was checked in the next stage using variance inflation factor (VIF). All variables included in the final models exhibited VIF values below the predefined threshold, indicating no evidence of problematic multicollinearity. Therefore, no variables were excluded on this basis. Finally, the selected variables were adjusted in the models. The predictor variables used in this study included maternal age at childbirth, maternal education, preceding pregnancy interval, access and exposure to the media, maternal pregnancy desire (wanted pregnancy when became pregnant), delivery type, place of delivery, history of terminated pregnancy, pregnancy outcome in terms of twin birth or single birth, decision-making autonomy. Although place of delivery and mode of delivery are related variables, they were retained simultaneously as they capture distinct dimensions of maternal healthcare utilization, namely access to facility-based care and type of obstetric intervention received [21,22]. Household socioeconomic status was measured using the wealth index, a composite indicator of cumulative living standards derived from household asset ownership through principal component analysis [23]. Region of residence was also included and categorized as Barisal, Chittagong, Dhaka, Mymensingh, Khulna, Rajshahi, Rangpur, and Sylhet. The definition of the predictor variables is available in the Supplementary Table 1 in S1 File.

### Statistical analyses

Descriptive statistics and percentages were used to summarize the characteristics of the respondents. In case of predictor variables, the missing values were directly reported, and no specific measures were taken to replace the missing values. However, in the case of outcome variables, the cases with missing values were excluded from the analysis. Multivariable binary logistic regression analyses were conducted to identify the determinants of the outcome variables. Five separate logistic regression models were developed for the five outcome variables: stillbirth, preterm birth, low birth weight, neonatal death, and adverse birth outcome. Variables with p-values < 0.25 in the bivariate analyses were considered for inclusion in the regression models [24]. Before finalizing the models, the underlying assumptions of logistic regression were assessed. Multicollinearity was evaluated using the VIF, with a VIF value exceeding 2 indicating potential multicollinearity [25]. The goodness-of-fit for the regression models was assessed using Pearson’s goodness-of-fit statistic. Associations were reported as adjusted odds ratios (AOR) with 95% confidence intervals (CI). However, the unadjusted models, model fits, and VIF values for adjusted models are given in the Supplementary Tables 2 and 3 in S1 File. All analyses were two-sided, with statistical significance defined as p < 0.05. To ensure national representativeness, all analyses were conducted in a weighted environment and addressed the complex survey design of the survey as suggested by DHS methodology [26]. Svyset command in stata was use to addressed the complex design where women individual weight factor provided in the BDHS dataset was used as [pw] along with strata and psu variables. All statistical analyses were performed using Stata, version 15 (StataCorp, College Station, TX 77845, USA).

### Ethics approval

This study used publicly available data from the DHS Program (www.dhsprogram.com), accessed upon request in accordance with the official data sharing policy. The survey authorities obtained all necessary ethical approvals and permissions prior to data collection. Therefore, no additional ethical approval was required for this secondary data analysis.

## Results

### Background characteristics of the study participants

The findings indicate that 54% of mothers completed secondary education, and household wealth was evenly distributed across quintiles (approximately 20% each) (Table 1). Nearly 18% of mothers reported a history of a terminated pregnancy. Around two-thirds of deliveries (64.5%) occurred in healthcare facilities, with 45% being cesarean sections. The vast majority of births (99%) were single-child deliveries. About 81% of pregnancies were wanted at conception, and 58% of mothers had media exposure. A preceding pregnancy interval of ≥ 2 years was reported by 56% of mothers. Most mothers (61%) gave birth for the first time between the ages of 20 and 30 years, and 74% had decision-making autonomy. Regional distribution varied, with the highest proportion from Dhaka (25%) and the lowest from Sylhet (6.3%) followed by Barishal (6.5%) (Table 1).

**Table 1:** Sociodemographic characteristics of the study population (N = 10300).

Characteristics	Frequency (n)	Percentage (%)
**Maternal education**		
No education	591	5.7
Primary	2303	22.3
Secondary	5599	54.2
Higher secondary and above	1836	17.8
**Household wealth index**		
Poorest	2161	20.9
Poorer	2099	20.3
Middle	2103	20.4
Richer	2035	19.7
Richest	1932	18.7
**History of terminated pregnancy**		
Yes	1873	18.1
No	8457	81.9
**Place of delivery (N = 5104)**		
Home	1812	35.5
Hospital and clinic	3291	64.5
**Delivery by caesarean section (N = 5090)**		
Yes	2273	44.6
No	2817	55.4
**Pregnancy outcome**		
Single child	10235	99.1
Twin child	95	0.9
**Maternal pregnancy desire (N = 5104)**		
Yes	4131	80.9
No	972	19.1
**Exposure to media**		
Yes	5940	57.5
No	4389	42.5
**Preceding pregnancy interval**		
First Pregnancy	3354	32.6
Less than 2 years	1155	11.2
2 years or more	5791	56.2
**Maternal age at 1**^**st**^ **childbirth**		
20 years or less	2311	22.4
20-30 years	6297	61
More than 30 years	1721	16.7
**Decision-making autonomy (N = 6776)**		
Yes	4998	73.8
No	1778	26.2
**Division**		
Barishal	667	6.5
Chattogram	2176	21.1
Dhaka	2604	25.2
Khulna	1086	10.5
Mymensingh	872	8.4
Rajshahi	1110	10.7
Rangpur	1167	11.3
Sylhet	647	6.3

### Prevalence of adverse birth outcomes

**Fig 2:**
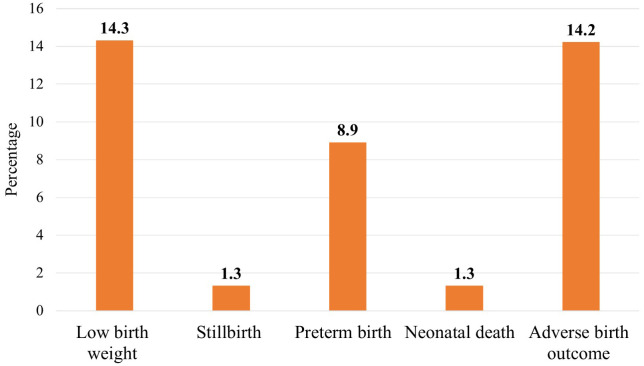
Prevalence of low birth weight, stillbirth, preterm birth, neonatal death, and composite adverse birth outcome.

Low birth weight was the most prevalent adverse birth outcome, affecting 14.3% of births, followed by preterm birth at 8.9%. Stillbirth and neonatal death each occurred in 1.3% of cases. The composite outcome was therefore largely driven by low birth weight and preterm birth events. Overall, 14.2% of births were associated with at least one adverse outcome (Fig 2).

### Risk factors of preterm birth and low birth weight

Women belonging to a household with a middle wealth index experienced significantly lower odds of preterm birth (AOR: 0.64, 95% CI: 0.43–0.96), while being in a richer household was linked to reduced odds of low birth weight (AOR: 0.66, 95% CI: 0.45–0.98). Delivery in a hospital or clinic was associated with significantly lower risk of low birth weight (AOR: 0.64, 95% CI: 0.43–0.95) compared to delivery take place in home. Cesarean delivery increased the likelihood of preterm birth (AOR: 1.52, 95% CI: 1.10–2.11). Twin births were strongly associated with higher odds of low birth weight (AOR: 6.80, 95% CI: 2.68–17.24), while having a significantly lower odds of preterm birth (AOR: 0.13, 95% CI: 0.02–0.98). Maternal desire for pregnancy was associated with lower odds of both preterm birth (AOR: 0.66, 95% CI: 0.50–0.86) and low birth weight (AOR: 0.67, 95% CI: 0.50–0.89). Regional variations were observed, with lower odds of low birth weight in Khulna (AOR: 0.56, 95% CI: 0.34–0.93) and Mymensingh (AOR: 0.58, 95% CI: 0.35–0.97) compared to Barishal (Table 2).

**Table 2:** Factors associated with preterm birth and low birth weight.

Characteristics	Preterm birth n (%)	AOR (95% CI)	p-value	Low birth weight n (%)	AOR (95% CI)	p-value
**Maternal education**						
No education	29 (5.1)	1		25 (22.2)	1	
Primary	170 (7.5)	1.48 (0.68, 3.23)	0.321	84 (16.4)	0.76 (0.44, 1.31)	0.320
Secondary	501 (9.1)	1.95 (0.93, 4.10)	0.076	252 (13.9)	0.71 (0.43, 1.17)	0.177
Higher secondary and above	211 (11.6)	2.06 (0.94, 4.53)	0.072	100 (12.7)	0.69 (0.39, 1.21)	0.192
**Household wealth index**						
Poorest	145 (6.8)	1		77 (18.5)	1	
Poorer	159 (7.7)	0.78 (0.52, 1.15)	0.211	84 (14.4)	0.78 (0.51, 1.19)	0.255
Middle	186 (8.9)	0.64 (0.43, 0.96)	0.033	88 (13.1)	0.70 (0.47, 1.04)	0.076
Richer	193 (9.6)	0.75 (0.51, 1.11)	0.151	95 (12.4)	0.66 (0.45, 0.98)	0.038
Richest	229 (11.9)	0.82 (0.53, 1.25)	0.352	118 (14.7)	0.83 (0.54, 1.28)	0.408
**History of terminated pregnancy**						
Yes	176 (10.2)	1.05 (0.76, 1.46)	0.746	98 (16.2)	1.12 (0.79, 1.59)	0.531
No	735 (8.7)	1		363 (13.9)	1	
**Place of delivery**						
Home	113 (6.3)	1		58 (22.5)	1	
Hospital and clinic	330 (10.2)	1.15 (0.78, 1.68)	0.488	404 (13.6)	0.64 (0.43, 0.95)	0.027
**Delivery by caesarean section**						
Yes	253 (11.2)	1.52 (1.10, 2.11)	0.012	269 (12.6)	0.85 (0.64, 1.13)	0.261
No	188 (6.8)	1		191 (17.6)	1	
**Pregnancy outcome**						
Single child	897 (8.9)	1		443 (13.9)	1	
Twin child	15 (16.5)	0.13 (0.02, 0.98)	0.048	19 (50)	6.80 (2.68, 17.24)	< 0.001
**Maternal pregnancy desire**						
Yes	336 (8.3)	0.66 (0.50, 0.86)	0.003	354 (13.2)	0.67 (0.50, 0.89)	0.007
No	107 (11.2)	1		108 (19.3)	1	
**Exposure to media**						
Yes	610 (10.4)	1.26 (0.97, 1.63)	0.077	275 (13)	0.90 (0.70, 1.15)	0.379
No	302 (7)	1		187 (16.6)	1	
**Preceding pregnancy interval**						
First pregnancy	262 (7.9)	0.83 (0.63, 1.09)	0.181	154 (12.6)	0.95 (0.71, 1.28)	0.747
Less than 2 years	129 (11.4)	1.13 (0.79, 1.62)	0.488	63 (17.2)	1.15 (0.77, 1.70)	0.499
2 years or more	514 (9)	1		238 (14.6)	1	
**Division**						
Barishal	51 (7.8)	1		28 (16.7)	1	
Chattogram	190 (8.9)	1.46 (0.94, 2.26)	0.093	112 (18.2)	1.18 (0.73, 1.90)	0.493
Dhaka	283 (11)	1.40 (0.90, 2.18)	0.138	155 (17.5)	1.15 (0.72, 1.82)	0.561
Khulna	102 (9.5)	1.17 (0.74, 1.85)	0.491	38 (9)	0.56 (0.34, 0.93)	0.024
Mymensingh	55 (6.4)	0.86 (0.50, 1.47)	0.572	24 (10.3)	0.58 (0.35, 0.97)	0.037
Rajshahi	126 (11.5)	1.27 (0.78, 2.04)	0.333	34 (9)	0.61 (0.36, 1.03)	0.064
Rangpur	76 (6.6)	0.68 (0.39, 1.17)	0.167	42 (11.3)	0.60 (0.36, 1.02)	0.058
Sylhet	29 (4.6)	0.74 (0.42, 1.32)	0.316	29 (18.2)	1.12 (0.68, 1.85)	0.645

**Note:** AOR: Adjusted odds ratio; CI: Confidence interval; n (%) refers to the number and percentage of births experiencing the outcome within each category.

### Risk factors of stillbirth and neonatal death

Cesarean delivery was linked to lower odds of both stillbirth (AOR: 0.39, 95% CI: 0.21–0.71) and neonatal death (AOR: 0.31, 95% CI: 0.14–0.68). Additionally, twin births were associated with significantly higher odds of stillbirth (AOR: 5.54, 95% CI: 1.10–27.74) and neonatal death (AOR: 5.90, 95% CI: 1.79–19.48) compared to single births. Besides meternal pregnancy desire was associated with lower risk of neonatal dealth (AOR: 0.50, 95% CI: 0.28–0.90) (Table 3).

**Table 3:** Factors associated with stillbirth and neonatal death.

Characteristics	Stillbirth n (%)	AOR (95% CI)	p-value	Neonatal death n (%)	AOR (95% CI)	p-value
**Maternal education**						
No education	13 (2.1)	1				
Primary	34 (1.5)	0.68 (0.24, 1.87)	0.449			
Secondary	75 (1.3)	0.79 (0.32, 1.95)	0.611			
Higher secondary and above	14 (0.8)	0.77 (0.24, 2.51)	0.665			
**Household wealth index**						
Poorest	36 (1.6)	1		42 (2)	1	
Poorer	30 (1.4)	0.78 (0.37, 1.62)	0.501	30 (1.4)	0.62 (0.28, 1.36)	0.234
Middle	24 (1.1)	0.83 (0.41, 1.67)	0.600	29 (1.4)	0.58 (0.26, 1.29)	0.183
Richer	33 (1.6)	1.00 (0.48, 2.08)	0.990	16 (0.8)	0.46 (0.19, 1.16)	0.100
Richest	14 (0.7)	0.74 (0.30, 1.83)	0.515	16 (0.8)	0.48 (0.20, 1.16)	0.101
**Place of delivery**						
Home				32 (1.8)	1	
Hospital and clinic				37 (1.1)	1.44 (0.75, 2.76)	0.273
**Delivery by caesarean section**						
Yes	19 (0.8)	0.39 (0.21, 0.71)	0.002	14 (0.6)	0.31 (0.14, 0.68)	0.004
No	58 (2.1)	1		53 (1.9)	1	
**Pregnancy outcome**						
Single child	130 (1.3)	1		127 (1.3)	1	
Twin child	6 (6.3)	5.54 (1.10, 27.74)	0.037	7 (7.4)	5.90 (1.79, 19.4)	0.004
**Maternal pregnancy desire**						
Yes				43 (1.1)	0.50 (0.28, 0.90)	0.022
No				25 (2.6)	1	
**Preceding pregnancy interval**						
First pregnancy	41 (1.2)	1.47 (0.75, 2.86)	0.257	31 (0.9)	1.24 (0.62, 2.50)	0.543
Less than 2 years	24 (2.1)	1.91 (0.99, 3.68)	0.055	21 (1.8)	1.40 (0.65, 3.05)	0.392
2 years or more	70 (1.2)	1		79 (1.4)	1	
**Maternal age at 1**^**st**^ **childbirth**						
20 or less	34 (1.5)	1		21 (0.9)	1	
20-30	70 (1.1)	0.80 (0.46, 1.41)	0.443	83 (1.3)	1.97 (0.87, 4.47)	0.105
More than 30	32 (1.8)	1.22 (0.53, 2.82)	0.642	29 (1.7)	2.15 (0.74, 6.25)	0.160

AOR: Adjusted odds ratio; CI: Confidence interval; n (%) refers to the number and percentage of births experiencing the outcome within each category.

### Risk factors of adverse birth outcome

Women from households with middle wealth index (AOR: 0.72, 95% CI: 0.54–0.95) experienced significantly lower odds of adverse birth outcomes compared to those from the poorest households (Table 4). History of terminated pregnancy was associated with 1.75 times higher odds of adverse birth outcomes (AOR: 1.75, 95% CI: 1.36–2.23). Women who delivered in a hospital or clinic experienced more than twice the odds of adverse birth outcomes (AOR: 2.13, 95% CI: 1.65–2.74) compared to those who delivered at home. Twin births had nearly four times higher odds of adverse birth outcomes (AOR: 4.10, 95% CI: 1.90–8.86) than single births. Wanted pregnancies were significantly associated with lower odds of adverse birth outcomes (AOR: 0.58, 95% CI: 0.47–0.70), and decision-making autonomy was protective, reducing the odds of adverse birth outcomes (AOR: 0.81, 95% CI: 0.67–0.97). Regionally, women from Mymensingh (AOR: 0.67, 95% CI: 0.46–0.96) and Rangpur (AOR: 0.66, 95% CI: 0.44–0.99) experienced significantly lower odds of adverse birth outcomes compared to those in Barishal (Table 4).

**Table 4:** Factors associated with adverse birth outcome.

Characteristics	Adverse birth outcome n (%)	AOR (95% CI)	p-value
**Maternal education**			
No education	72 (12.1)	1	
Primary	289 (12.5)	0.93 (0.62, 1.40)	0.729
Secondary	803 (14.3)	1.10 (0.75, 1.61)	0.627
Higher secondary and above	304 (16.6)	1.17 (0.76, 1.81)	0.468
**Household wealth index**			
Poorest	263 (12.2)	1	
Poorer	264 (12.6)	0.76 (0.58, 1.01)	0.063
Middle	301 (14.3)	0.72 (0.54, 0.95)	0.021
Richer	304 (15)	0.77 (0.59, 1.00)	0.052
Richest	336 (17.4)	0.87 (0.63, 1.19)	0.368
**History of terminated pregnancy**			
Yes	389 (20.8)	1.75 (1.36, 2.23)	< 0.001
No	1078 (12.7)	1	
**Place of delivery**			
Home	204 (11.3)	1	
Hospital and clinic	704 (21.4)	2.13 (1.65, 2.74)	< 0.001
**Delivery by caesarean section**			
Yes	475 (20.9)	0.98 (0.79, 1.22)	0.872
No	427 (15.1)	1	
**Pregnancy outcome**			
Single child	1426 (13.9)	1	
Twin child	41 (43.7)	4.10 (1.90, 8.86)	< 0.001
**Maternal pregnancy desire**			
Yes	685 (16.6)	0.58 (0.47, 0.70)	< 0.001
No	222 (22.9)	1	
**Exposure to media**			
Yes	923 (15.5)	1.13 (0.95, 1.36)	0.171
No	544 (12.4)	1	
**Preceding pregnancy interval**			
First pregnancy	443 (13.2)	1.07 (0.82, 1.39)	0.614
Less than 2 years	213 (18.4)	1.01 (0.77, 1.33)	0.932
2 years or more	797 (13.8)	1	
**Maternal age at 1**^**st**^ **childbirth**			
20 or less	332 (14.4)	1	
20-30	867 (13.8)	0.90 (0.72, 1.13)	0.353
More than 30	268 (15.6)	0.95 (0.68, 1.34)	0.786
**Decision-making autonomy**			
Yes	786 (15.7)	0.81 (0.67, 0.97)	0.023
No	305 (17.2)	1	
**Division**			
Barishal	89 (13.3)	1	
Chattogram	320 (14.7)	1.09 (0.76, 1.57)	0.640
Dhaka	434 (16.7)	1.09 (0.76, 1.55)	0.637
Khulna	148 (13.6)	0.75 (0.50, 1.12)	0.156
Mymensingh	98 (11.3)	0.67 (0.46, 0.96)	0.028
Rajshahi	172 (15.5)	0.82 (0.55, 1.22)	0.329
Rangpur	132 (11.3)	0.66 (0.44, 0.99)	0.045
Sylhet	75 (11.5)	0.89 (0.60, 1.32)	0.572

**Note:** AOR: Adjusted odds ratio; CI: Confidence interval; n (%) refers to the number and percentage of births experiencing the outcome within each category.

## Discussion

This study explored the prevalence and determinants of adverse birth outcomes in Bangladesh using nationwide survey data. The findings indicate that LBW was the most common adverse outcome, affecting 14.3% of births, followed by preterm birth (8.9%), while stillbirth (1.3%) and neonatal death (1.3%) were the least reported. Overall, 14.2% of births were associated with at least one adverse outcome. Key contributing factors included maternal characteristics (decision-making autonomy), pregnancy-related factors (unwanted pregnancy, history of terminated pregnancy, and pregnancy outcome), delivery-related factors (caesarean section, place of delivery), and sociodemographic determinants (household wealth index and geographic region).

### Low birth weight

Our study found that approximately one in every seven births in Bangladesh is affected by LBW, a finding that is consistent with those reported in earlier studies in Bangladesh [22,23]. Compared to other countries, the prevalence of LBW in Bangladesh is similar to that of Ethiopia [27], slightly higher than in Nepal [28], and slightly lower than in India [29].

In line with the previous studies, this study found that children from the poorest households were more likely to be born with LBW [30,31,28,29]. Several pathways can explain the association of wealth status and the birth of an LBW child. Women from households with limited financial resources often have reduced access to nutritious food, are more likely to be malnourished, which in turn leads to LBW children [32]. Moreover, these women are less likely to receive adequate care during pregnancy, which collectively increases the risk of delivering an LBW infant with other factors [33].

This study found a significant association between the place of delivery and the risk of LBW, with hospital or clinic deliveries being associated with a substantially lower risk of LBW compared to home deliveries. This finding aligns with earlier research indicating that the prevalence of LBW was significantly higher among infants born at home than those born in health facilities [34]. Birth weight is primarily determined by gestational age and intrauterine growth [35]. The observed association between place of delivery and the risk of LBW may largely reflect differences in underlying maternal health and socioeconomic conditions that influence intrauterine growth rather than the act of delivery itself. Women who deliver in hospitals or clinics are more likely to be of higher socioeconomic status, with better access to adequate nutrition, regular and quality antenatal care, and overall health services throughout pregnancy [36,37], factors that reduce the risk of intrauterine growth restriction, a major contributor to LBW.

Similar to earlier studies, this study found that twin births are more likely to result in LBW compared to singleton births [28,38]. This can be attributed to the higher risk of prematurity associated with twin pregnancies [39], as premature birth is a major contributing factor to LBW and its related complications [40]. In this study, LBW was more common among infants born to women with unwanted pregnancies. The findings of previous research also bolster our findings [41,42]. Women with unwanted pregnancies are substantially less likely to attend adequate antenatal care, including regular ANC visits [43] and iron–folic acid supplementation [44]. Consequently, they face greater risks of inappropriate gestational weight gain and higher cesarean section rates [45], as well as increased likelihoods of prematurity [46] and preeclampsia [41], all of which contribute to a higher incidence of LBW deliveries.

### Preterm birth

In our study, the prevalence of preterm birth was 8.9%, which is notably lower than the 14.6% reported in a study conducted in the Rajshahi division [9], and the 21.7% observed among births in urban slum areas [47]. This difference may be attributed to the fact that our findings are based on a nationally representative dataset, offering a broader population perspective. In contrast, the higher rates reported in slum areas can be attributed to the fact that mothers in slum areas face greater socioeconomic disadvantages, limited access to quality healthcare, and inadequate maternal support services—all of which are known contributors to increased preterm birth rates [48].

Similar to earlier research, this study found that women from wealthier households had significantly lower odds of giving preterm birth compared to those from poorer households [49]. This finding reinforces the established link between socioeconomic status and maternal as well as child health outcomes. Women from wealthier households are more likely to access quality antenatal care, maintain better nutrition, and face fewer stressors, reducing the risk of preterm birth. In contrast, those from poorer households often encounter limited healthcare, higher infection rates, and greater physical strain, increasing the risk of adverse outcomes [50].

This study identified a significant association between cesarean section (C-section) delivery and preterm birth, corroborating findings from earlier research [51]. The observed association between cesarean delivery and preterm birth may be partially explained by the timing and clinical indications for cesarean procedures. Specifically, cesarean sections performed before or during early labor are frequently medically indicated due to maternal or fetal complications, which themselves increase the likelihood of preterm birth [52]. Thus, obstetric complications play an important role in shaping this association [51]. However, because cesarean delivery inherently determines the timing of birth, procedures performed without strong medical indication, particularly elective or early-term C-sections, may also contribute to deliveries occurring earlier than would otherwise have occurred [53]. These findings underscore the importance of distinguishing medically necessary cesarean deliveries from potentially avoidable procedures when interpreting their relationship with preterm birth.

This study found that twin births were significantly associated with lower odds of preterm birth, which contrasts with the majority of existing literature that typically reports a higher risk of preterm birth in multiple pregnancies [54]. It likely reflects over-adjustment for delivery-related factors and instability due to the small number of multiple births rather than a true protective effect. Though in some cases, twins may receive more frequent antenatal monitoring and earlier medical attention, leading to timely interventions that help prolong gestation. Additionally, if gestational age is underreported or inaccurately assessed—particularly in home births or low-resource settings—this may lead to misclassification, affecting observed associations. It is also possible that, in this study setting, singleton pregnancies with complications were more likely to result in preterm birth, skewing the comparative risk profile. Overall, while twin pregnancies were associated with several adverse birth outcomes in this study, the inverse association observed for preterm birth suggests heterogeneity in their effects across outcomes and should be interpreted with caution, as it does not indicate a protective role.

Consistent with previous research, this study found that women with a wanted pregnancy had lower odds of experiencing preterm birth [55]. This association may be attributed to the fact that women with unwanted pregnancies are often less emotionally prepared and may delay seeking antenatal care [44]. They are also less likely to follow nutritional or medical guidance and more likely to experience stress and anxiety, all of which can negatively affect maternal and fetal health. Limited family or partner support in such cases may further reduce access to timely and adequate care, increasing the risk of complications such as preterm birth.

### Stillbirth

Our study found that the prevalence of stillbirth was 1.3%, which is considerably lower than the pooled prevalence (2.8%) reported in a previous study in Bangladesh [56]. This difference may be largely explained by the time gap between the studies, as the earlier research was conducted much earlier. Over time, there have been significant improvements in maternal and child health services in Bangladesh [57,58], which likely contributed to the lower stillbirth prevalence observed in our study. Compared to other countries, the stillbirth prevalence in our study is also lower than that reported in India (2.5%), Pakistan (5.6%), Guatemala (1.9%), and Africa (Zambia and Kenya) (2.1%) [59], suggesting that Bangladesh has made notable progress in addressing the factors associated with stillbirth.

In this study, we found that cesarean delivery was associated with lower odds of stillbirth, which contrasts with findings from earlier studies that reported a higher likelihood of stillbirth among cesarean deliveries [60,61]. This apparent protective association should be interpreted with caution, as it may reflect selection mechanisms, differences in case severity, or residual confounding rather than a true causal effect of cesarean delivery. In some cases, timely access to emergency obstetric care and appropriately indicated surgical intervention may contribute to improved fetal survival, particularly when complications are promptly recognized and managed. However, in settings with high cesarean section rates like Bangladesh [62], this statistical association should not be interpreted as evidence that increasing cesarean use would reduce stillbirth at the population level. Instead, the findings underscore the importance of ensuring that cesarean deliveries are performed based on clear medical indications and within a high-quality emergency obstetric care framework.

Consistent with previous research, this study found that twin births were associated with significantly higher odds of stillbirth [63]. This increased risk may be attributed to a higher likelihood of complications in multiple pregnancies, such as preterm birth, intrauterine growth restriction, placental insufficiency, and delivery-related challenges [64,65], all of which contribute to poorer perinatal outcomes compared to singleton pregnancies.

### Neonatal death

In our study, the prevalence of neonatal death was 1.3% (13 per 1,000 live births), which is notably lower than the national neonatal mortality rate of 31.9 per 1,000 live births reported in the 2017 BDHS [66]. However, recent data suggest a declining trend in national neonatal mortality, likely due to improvements in healthcare access, increased community awareness, and effective government policies and interventions targeting maternal and newborn health [66]. When compared to a community-based cohort study conducted in rural sub-districts of Sylhet Division, which reported a neonatal mortality rate of 43.4 per 1,000 live births [67], our findings are substantially lower. The higher mortality rate observed in that rural setting may be attributed to factors such as traditional birth practices, lower maternal education levels, and limited access to skilled birth attendants and essential neonatal care services [68].

In this study, cesarean delivery was associated with lower odds of neonatal death, a finding that contrasts with previous studies which reported higher neonatal mortality among cesarean births [69,70]. While cesarean sections are often performed in response to obstetric complications, timely access to appropriately indicated surgical intervention and high-quality neonatal care in facility settings may contribute to improved survival among selected cases. Additionally, gestational age may also influence this relationship, as operative delivery can determine the timing of birth and, in some instances, prevent prolonged labor or fetal compromise [71]. However, in this case, this finding underscores the need for cesarean deliveries to be based on clear medical indications within a strong continuum of maternal and newborn care.

Similar to previous research, we found that twin births had significantly higher odds of neonatal mortality compared to singleton births [67]. This increased risk may be attributed to the higher likelihood of preterm birth, low birth weight, and increased incidence of antenatal and intrapartum complications commonly observed in multiple pregnancies. The convergence of these risk factors likely contributes to the elevated neonatal mortality among twins [72]. This study also found that intended pregnancies had significantly lower odds of neonatal mortality than unintended pregnancies, aligning with prior evidence [73]. This may be attributed to better antenatal care access, health-seeking behaviors, or psychosocial advantages among mothers with planned pregnancies.

### Adverse birth outcome

In this study, the overall prevalence of composite adverse birth outcomes was 14.2%. While directly comparable data from Bangladesh are lacking, our findings align closely with rates reported in India (12.2%) [74] and South Africa (14.6%) [75], though higher prevalence was observed in Nepal (20.9%) and Ethiopia (26.8%) [76]. These variations may reflect differences in healthcare infrastructure, maternal health services, socio-demographic factors, and outcome definition.

Consistent with previous research, this study found that women from wealthier households experienced significantly lower odds of adverse birth outcomes compared to those from the poorest households [7]. This may be attributed to better socio-economic conditions, which can facilitate timely health-seeking behaviors, improved maternal nutrition, greater awareness of pregnancy-related danger signs, and early initiation of antenatal care [7], all of which contribute to better birth outcomes.

Our study found that a history of terminated pregnancy was linked to increased odds of adverse birth outcomes, which was consistent with earlier research findings [77,78]. Surgical terminations, especially when inadequately managed, can cause cervical trauma, intrauterine adhesions, or infections such as pelvic inflammatory disease and endometritis, all of which may compromise uterine integrity and increase the risk of adverse outcomes in future pregnancies [79,80].

In contrast to earlier studies, our findings revealed that deliveries in hospitals or clinics were significantly associated with adverse birth outcomes [13,81]. This may be explained by the common practice in our country—especially in rural areas—where most deliveries are initially attempted at home due to limited awareness of potential complications during childbirth if proper care is not followed. Consequently, women are often referred to health facilities only after complications develop, which increases the risk of adverse outcomes.

Consistent with previous research, twin births were significantly associated with higher odds of composite adverse birth outcomes compared to singleton births [7]. This may be attributed to the higher incidence of complications such as pre-eclampsia and antepartum hemorrhage during twin pregnancies, which can contribute to unfavorable outcomes [7]. In line with prior studies, this study found that wanted pregnancies were significantly associated with reduced odds of adverse birth outcomes [42,82]. Wanted pregnancies are often associated with better maternal behaviors and access to prenatal care, which can contribute to healthier birth outcomes. Additionally, women with wanted pregnancies are more likely to seek early and regular antenatal care, avoid harmful substances, and experience lower stress levels—all factors that reduce the risk of adverse birth outcomes such as low birth weight or preterm birth [83].

In our study, maternal decision-making autonomy emerged as a protective factor, reducing the odds of adverse birth outcomes—aligning with findings from previous research [84,85]. Greater autonomy empowers women to make informed decisions about their health, enabling timely access to prenatal care, adherence to medical advice, and the adoption of healthier behaviors during pregnancy [86,87]. These factors collectively contribute to improved birth outcomes. In contrast, limited autonomy can hinder access to essential healthcare services and increase exposure to psychosocial stressors—both of which are well-documented risk factors for adverse outcomes.

### Policy implications and recommendations

This study underscores the need for targeted policies to reduce adverse birth outcomes in Bangladesh. Key findings suggest that addressing socioeconomic inequality is critical, as women from the poorest households consistently experienced higher risks of adverse birth outcomes. Expanding social protection and maternal health support in disadvantaged communities is essential. The study also highlights the importance of reproductive health services, including family planning and birth spacing, to reduce unintended pregnancies which was associated with adverse outcomes. Special attention should be given to women with a history of terminated pregnancy, through enhanced antenatal monitoring.

Women’s empowerment, particularly decision-making autonomy, emerged as a strong protective factor. Promoting gender equity and integrating empowerment strategies into health programs can enhance maternal health behaviors and outcomes. Although hospital and cesarean deliveries showed mixed associations, findings point to the need for timely, high-quality facility-based care. Strengthening referral systems and emergency obstetric services is crucial to prevent complications, especially in rural areas. Finally, twin pregnancies and high-risk cases should be prioritized in clinical protocols and health programming. Tailored interventions for such groups can reduce the burden of adverse birth outcomes.

### Strengths and limitations

This study has several strengths and a few limitations. The present study is the first of its kind in Bangladesh to investigate the prevalence and determinants of a composite indicator of adverse birth outcomes, along with four individual outcome variables that comprise this composite score. A significant strength of our study is the utilization of nationally representative survey data. However, there are some methodological limitations. Since this is a cross-sectional study, we cannot establish causality between adverse birth outcomes and the identified factors. Additionally, because the analysis is secondary in nature, some important factors may remain unexplored. To minimize recall bias, we analyzed the most recent birth that occurred within the five years preceding the survey. Despite this, the possibility of bias may still exist. The study utilized complex survey design of BDHS and employs appropriate statistical models to assess the association between predictors and outcomes, which ensures representativeness and generalisability. However, the analysis of low birth weight was restricted to births with reported birth weight, which substantially reduced the analytic sample and complete-case analysis may have introduced selection bias, as 68.9 percent of infants were not weighed at birth. Births with recorded birth weight differed systematically from those without measurements. Mothers of infants with available birth weight data were significantly more likely to have secondary or higher education, belong to higher wealth quintiles, and deliver in health facilities compared with mothers whose infants were not weighed at birth (Supplementary Table 5 in S1 File). These patterns indicate that the LBW analytic sample represents a more socioeconomically advantaged subset of the study population. This suspected selection bias may limit the generalizability of the LBW estimates, which should therefore be interpreted with caution. Also missing data in predictor variables were an issue in this analysis as DHS survey methodology allows to collect data from one woman in a household. Another issue is that the composite adverse birth outcome includes events with distinct etiologies and conditional relationships and is largely driven by common outcomes, such as low birth weight and preterm birth, which may obscure associations with rarer outcomes. To ensure robustness of the conclusion, we therefore examined each outcome separately. Despite these limitations, this study provides valuable insights into the maternal and household factors associated with adverse birth outcomes, which could inform policymaking aimed at reducing the incidence of such outcomes in the context of Bangladesh.

## Conclusion

This study underscores the substantial burden of adverse birth outcomes in Bangladesh, with LBW being the most prevalent concern. The findings reveal that these outcomes stem from a complex interplay of maternal, obstetric, delivery-related, and sociodemographic determinants. Key modifiable factors—such as maternal education, decision-making autonomy, and healthcare access—emerge as critical intervention points. To mitigate adverse birth outcomes and enhance neonatal health, targeted policies and programs must prioritize maternal health services, family planning education, and equitable access to quality prenatal and delivery care. Addressing these underlying determinants will be essential for improving birth outcomes and ensuring healthier futures for mothers and newborns across Bangladesh.

## Supporting information

S1 File Supplementary Materials. (DOCX)
