# Supplementary material for: Prevalence and risk factors of adverse birth outcomes in Bangladesh: Insight from a nationwide survey
Source: PLoS One. 2026 Jun 17;21(6):e0351676. doi: 10.1371/journal.pone.0351676 (PMC13274831; doi:10.1371/journal.pone.0351676)
Supplement: S1 File — (DOCX) [file pone.0351676.s001.docx]

**Supplementary Table 1: Operational definition of variables**

| **Variable Name** | **Brief definition** |
| --- | --- |
| ***Outcome variables*** | |
| **Preterm birth** | A preterm birth is a live birth that takes place before 37 full weeks of gestation, or 9 months of pregnancy. |
| **Low birth weight** | A live birth where the recorded birth weight is less than 2500 grams. |
| **Stillbirth** | A stillbirth is a pregnancy outcome where the baby was born dead after 28 completed weeks (7 months) of gestation. |
| **Neonatal death** | A neonatal death is the death of a live-born infant within the first 28 days of life. |
| **Adverse birth outcome** | An adverse birth outcome is the presence of at least one of the following conditions: low birth weight, preterm birth, stillbirth, or neonatal death. |
| ***Predictor variables*** | |
| **Division** | Division refers to the highest administrative region in Bangladesh. The eight divisions are Barishal, Chattogram, Dhaka, Khulna, Mymensingh, Rajshahi, Rangpur, and Sylhet. |
| **Maternal education** | Maternal education indicates the highest level of formal education achieved by the mother. This variable is classified into 4 categories i.e. no education, primary education, secondary education, and higher education. |
| **Wealth index** | A composite measure of household socio-economic status based on assets and living conditions, categorized into quintiles from poorest to richest. |
| **History terminated pregnancy** | Pregnancies that ended in miscarriage, abortion, or other types of pregnancy termination; a dichotomous variable with yes and no categories. |
| **Place of delivery** | The location where a woman gave birth is categorized into two groups: home and hospital/clinic (including all other places except home). |
| **Delivery by caesarean section** | The delivery method was categorized as either a cesarean section or not, creating a dichotomous variable with "yes" and "no" options. |
| **Pregnancy outcome** | Whether the pregnancy outcome was a twin birth or a singular birth; categorized as two group i.e. twin birth and single birth. |
| **Maternal pregnancy desire** | Maternal desire to the pregnancy (mother wanted pregnancy when become pregnant); dichotomous variable with yes and no categories. |
| **Access & exposure to the media** | Weekly minimum access and exposure to at least one of three mass media (TV, Radio, and Newspaper) at least once a week; this is a dichotomous variable with yes or no categories. |
| **Preceding pregnancy interval** | The preceding pregnancy interval was categorized according to the international guidelines provided by WHO and UNICEF, which recommend a spacing of 24 months or two years. First pregnancies were treated separately. |
| **Mother's age at childbirth** | Maternal age at childbirth was categorized into three groups using a 10-year interval to separate the optimum maternal ages for pregnancy. |
| **Decision-making autonomy** | Explain whether the mother has minimum participation (alone or with another family member) in decision-making related to health care; dichotomous variable with yes or no categories. |

**Supplementary Table 2: Unadjusted binary logistic regression models**

| **Variables** | **Low birth weight** | | **Preterm birth** | | **Neonatal death** | | **Stillbirth** | | **Adverse birth outcome** | |
| --- | --- | --- | --- | --- | --- | --- | --- | --- | --- | --- |
|  | COR (95%CI) | P-value | COR (95%CI) | P-value | COR (95%CI) | P-value | COR (95%CI) | P-value | COR (95%CI) | P-value |
| Maternal education | | | | | | | | | | |
| No education | 1 |  | 1 |  | 1 |  | 1 |  | 1 |  |
| Primary | 0.69 (0.39, 1.21) | 0.197 | 1.51 (0.94, 2.43) | 0.085 | 0.74 (0.34, 1.63) | 0.456 | 0.68 (0.33, 1.41) | 0.302 | 1.04 (0.75, 1.44) | 0.819 |
| Secondary | 0.57 (0.34, 0.95) | 0.033 | 1.86 (1.20, 2.89) | 0.005 | 0.67 (0.32, 1.40) | 0.287 | 0.62 (0.32, 1.22) | 0.165 | 1.21 (0.90, 1.62) | 0.198 |
| Higher | 0.51 (0.30, 0.87) | 0.014 | 2.45 (1.56, 3.82) | 0.000 | 0.43 (0.17, 1.05) | 0.063 | 0.36 (0.16, 0.80) | 0.013 | 1.44 (1.06, 1.95) | 0.020 |
| Household wealth index | | | | | | | | | | |
| Poorest | 1 |  | 1 |  | 1 |  | 1 |  | 1 |  |
| Poorer | 0.74 (0.50, 1.11) | 0.144 | 1.14 (0.88, 1.46) | 0.317 | 0.72 (0.42, 1.24) | 0.237 | 0.85 (0.52,1.40) | 0.527 | 1.04 (0.85, 1.27) | 0.719 |
| Middle | 0.66 (0.46, 0.96) | 0.031 | 1.35 (1.05, 1.73) | 0.021 | 0.71 (0.41, 1.21) | 0.206 | 0.68 (0.4,1.14) | 0.143 | 1.21 (0.99, 1.46) | 0.059 |
| Richer | 0.62 (0.43, 0.89) | 0.010 | 1.46 (1.14, 1.87) | 0.003 | 0.39 (0.18, 0.84) | 0.015 | 0.97 (0.6,1.56) | 0.897 | 1.27 (1.05, 1.53) | 0.012 |
| Richest | 0.76 (0.52, 1.10) | 0.142 | 1.86 (1.44, 2.40) | 0.000 | 0.41 (0.22, 0.78) | 0.007 | 0.44 (0.24,0.82) | 0.010 | 1.52 (1.24, 1.86) | 0.000 |
| History of terminated pregnancy | | | | | | | | | | |
| Yes | 1.20 (0.91, 1.59) | 0.205 | 1.19 (0.97, 1.46) | 0.098 | 0.83 (0.49, 1.41) | 0.489 |  |  | 1.80 (1.54, 2.10) | 0.000 |
| No | 1 |  | 1 |  | 1 |  |  |  | 1 |  |
| Place of delivery | | | | | | | | | | |
| Home | 1 |  | 1 |  | 1 |  | 1 |  | 1 |  |
| Hospital and clinic | 0.54 (0.37, 0.78) | 0.001 | 1.69 (1.30, 2.20) | 0.000 | 0.63 (0.37, 1.06) | 0.082 | 1.26 (0.74, 2.15) | 0.392 | 2.14 (1.75, 2.63) | 0.000 |
| Delivery by caesarean section | | | | | | | | | | |
| Yes | 0.68 (0.52, 0.87) | 0.002 | 1.73 (1.38, 2.18) | 0.000 | 0.32 (0.16, 0.64) | 0.001 | 0.40 (0.23, 0.68) | 0.001 | 1.48 (1.24, 1.76) | 0.000 |
| No | 1 |  | 1 |  | 1 |  | 1 |  | 1 |  |
| Pregnancy outcome | | | | | | | | | | |
| Single child | 1 |  | 1 |  | 1 |  | 1 |  | 1 |  |
| Twin child | 6.23 (2.99, 2.96) | 0.000 | 2.03 (1.09, 3.80) | 0.026 | 6.26 (2.94, 3.34) | 0.000 | 5.26 (2.22, 12.46) | 0.000 | 4.80 (3.04, 7.55) | 0.000 |
| Wanted pregnancy when became pregnant | | | | | | | | | | |
| Yes | 0.64 (0.48, 0.85) | 0.002 | 0.71 (0.55, 0.92) | 0.011 | 0.40 (0.22, 0.71) | 0.002 | 1.01 (0.57, 1.80) | 0.962 | 0.67 (0.55, 0.81) | 0.000 |
| No | 1 |  | 1 |  | 1 |  | 1 |  | 1 |  |
| Exposure to media | | | | | | | | | | |
| Yes | 0.75 (0.60, 0.95) | 0.016 | 1.54 (1.29, 1.85) | 0.000 | 0.91 (0.61, 1.35) | 0.640 | 0.86 (0.60, 1.23) | 0.419 | 1.30 (1.14, 1.49) | 0.000 |
| No | 1 |  | 1 |  | 1 |  | 1 |  | 1 |  |
| Preceding pregnancy interval | | | | | | | | | | |
| First pregnancy | 0.85 (0.65, 1.10) | 0.208 | 0.87 (0.72, 1.05) | 0.150 | 0.67 (0.43, 1.07) | 0.094 | 1.01 (0.66, 1.56) | 0.952 | 0.95 (0.83, 1.09) | 0.497 |
| Less than 2 years | 1.22 (0.86, 1.72) | 0.265 | 1.30 (1.03, 1.65) | 0.028 | 1.33 (0.74, 2.42) | 0.342 | 1.74 (1.06, 2.85) | 0.029 | 1.41 (1.16, 1.72) | 0.001 |
| 2 years or more | 1 |  | 1 |  | 1 |  | 1 |  | 1 |  |
| Maternal age at 1st childbirth | | | | | | | | | | |
| 20 or less | 1 |  | 1 |  | 1 |  | 1 |  | 1 |  |
| 20-30 | 0.99 (0.77, 1.29) | 0.954 | 0.99 (0.81, 1.21) | 0.947 | 1.44 (0.88, 2.36) | 0.143 | 0.74 (0.46, 1.18) | 0.203 | 0.95 (0.82, 1.10) | 0.507 |
| More than 30 | 1.19 (0.81, 1.73) | 0.377 | 1.08 (0.83, 1.42) | 0.559 | 1.84 (0.97, 3.48) | 0.062 | 1.23 (0.72, 2.10) | 0.442 | 1.10 (0.90, 1.34) | 0.358 |
| Decision-making autonomy | | | | | | | | | | |
| Yes | 0.91 (0.71, 1.17) | 0.467 | 0.89 (0.71, 1.11) | 0.312 | 0.83 (0.48, 1.43) | 0.505 | 1.14 (0.70, 1.87) | 0.601 | 0.90 (0.76, 1.06) | 0.213 |
| No | 1 |  | 1 |  | 1 |  | 1 |  | 1 |  |
| Division | | | | | | | | | | |
| Barishal | 1 |  | 1 |  | 1 |  | 1 |  | 1 |  |
| Chattogram | 1.11 (0.70, 1.75) | 0.655 | 1.15 (0.83, 1.60) | 0.386 | 1.46 (0.69, 3.09) | 0.321 | 0.98 (0.42, 2.29) | 0.962 | 1.12 (0.88, 1.43) | 0.361 |
| Dhaka | 1.05 (0.68, 1.64) | 0.815 | 1.46 (1.09, 1.96) | 0.012 | 1.32 (0.61, 2.83) | 0.478 | 0.64 (0.26, 1.59) | 0.338 | 1.30 (1.02, 1.65) | 0.031 |
| Khulna | 0.49 (0.30, 0.81) | 0.005 | 1.25 (0.92, 1.70) | 0.157 | 0.80 (0.33, 1.92) | 0.612 | 1.07 (0.45, 2.54) | 0.886 | 1.02 (0.79, 1.33) | 0.860 |
| Mymensingh | 0.57 (0.34, 0.95) | 0.030 | 0.81 (0.55, 1.20) | 0.296 | 1.62 (0.68, 3.87) | 0.280 | 1.48 (0.62, 3.52) | 0.374 | 0.82 (0.63, 1.09) | 0.172 |
| Rajshahi | 0.49 (0.29, 0.84) | 0.010 | 1.54 (1.08, 2.19) | 0.016 | 1.37 (0.59, 3.15) | 0.465 | 1.13 (0.45, 2.83) | 0.787 | 1.19 (0.89, 1.58) | 0.239 |
| Rangpur | 0.63 (0.38, 1.05) | 0.077 | 0.84 (0.58, 1.21) | 0.342 | 2.01 (0.95, 4.25) | 0.066 | 0.82 (0.33, 2.05) | 0.673 | 0.83 (0.62, 1.12) | 0.222 |
| Sylhet | 1.11 (0.68, 1.81) | 0.675 | 0.57 (0.37, 0.86) | 0.008 | 2.16 (1.03, 4.56) | 0.042 | 1.06 (0.42, 2.68) | 0.907 | 0.85 (0.63, 1.14) | 0.277 |

**Supplementary Table 3: Variance inflation factors (VIFs) for variables included in the multivariable models**

| **Independent variables** | **Adverse birth outcome** | **Neonatal death** | **Stillbirth** | **Low birth weight** | **Preterm birth** |
| --- | --- | --- | --- | --- | --- |
| Maternal education | 1.271 |  | 1.251 | 1.204 | 1.268 |
| Household wealth index | 1.388 | 1.151 | 1.245 | 1.286 | 1.378 |
| History of terminated pregnancy | 1.05 |  |  | 1.068 | 1.051 |
| Place of delivery | 1.898 | 1.898 |  | 1.218 | 1.923 |
| Delivery by caesarean section | 1.842 | 1.855 | 1.129 | 1.239 | 1.869 |
| Pregnancy outcome | 1.006 | 1.005 | 1.005 | 1.007 | 1.005 |
| Wanted pregnancy when became pregnant | 1.032 | 1.029 |  | 1.028 | 1.026 |
| Exposure to media | 1.166 |  |  | 1.111 | 1.165 |
| Preceding pregnancy interval | 1.516 | 1.448 | 1.482 | 1.124 | 1.114 |
| Maternal age at 1st childbirth | 1.458 | 1.437 | 1.434 |  |  |
| Decision-making autonomy | 1.024 |  |  |  |  |
| Division | 1.022 |  |  | 1.03 | 1.023 |

**Supplementary Table 4: The percentage of missing data for outcome and predictor variables**

| **Variable name** | **Number of missing value** | **Percentage** |
| --- | --- | --- |
| Low Birth Weight | 7062 | 68.9 |
| Preterm Birth | 143 | 1.4 |
| Neonatal Death | 143 | 1.4 |
| Place of delivery | 5200 | 50.7 |
| Delivery by caesarean section | 5211 | 50.8 |
| Maternal pregnancy desire | 5200 | 50.7 |
| Decision-making autonomy | 3536 | 34.5 |

**Supplementary Table 5: Differences between births with and without recorded birth weight based on relevant socioeconomics**

|  | **Birth weight not recorded (%)** | **Birth weight recorded (%)** |
| --- | --- | --- |
| **Maternal education** |  |  |
| No education | 6.9 | 3.2 |
| Primary | 25.7 | 16.2 |
| Secondary | 51.6 | 54.6 |
| Higher | 15.9 | 26.1 |
| **Place of delivery** |  |  |
| Home | 82.5 | 7.8 |
| Hospital and clinic | 17.5 | 92.2 |
| **C-section delivery** |  |  |
| No | 92.0 | 33.1 |
| Yes | 8.0 | 66.9 |
| **Wealth quintile** |  |  |
| Poorest | 25.0 | 13.0 |
| Poorer | 20.7 | 17.4 |
| Middle | 19.5 | 19.9 |
| Richer | 18.0 | 23.4 |
| Richest | 16.7 | 26.3 |
