# Supplementary material for: Prevalence and risk factors of adverse birth outcomes in Bangladesh: Insight from a nationwide survey
Source: PLoS One. 2026 Jun 17;21(6):e0351676. doi: 10.1371/journal.pone.0351676 (PMC13274831; doi:10.1371/journal.pone.0351676)
Supplement: Attachment — Submitted filename: renamed_08b74.docx [file pone.0351676.s002.docx]

**Response to Editor’s comments**

**Comment 1:** Please ensure that your manuscript meets PLOS ONE's style requirements, including those for file naming. The PLOS ONE style templates can be found at

<https://journals.plos.org/plosone/s/file?id=wjVg/PLOSOne_formatting_sample_main_body.pdf>  and <https://journals.plos.org/plosone/s/file?id=ba62/PLOSOne_formatting_sample_title_authors_affiliations.pdf>

**Author response:** Thank you for your instruction. We have carefully reviewed the manuscript and ensured that it now fully complies with PLOS ONE’s style requirements, including the prescribed formatting and file naming conventions. The manuscript has been revised using the provided PLOS ONE style templates.

**Comment 2:** Thank you for stating the following in the Acknowledgments Section of your manuscript:

“This research received no specific grant from any funding agency in the public, commercial or not-for-profit sectors.”

We note that you have provided funding information that is not currently declared in your Funding Statement. However, funding information should not appear in the Acknowledgments section or other areas of your manuscript. We will only publish funding information present in the Funding Statement section of the online submission form.

Please remove any funding-related text from the manuscript and let us know how you would like to update your Funding Statement. Currently, your Funding Statement reads as follows:

“The author(s) received no specific funding for this work.”

Please include your amended statements within your cover letter; we will change the online submission form on your behalf.

**Author response:** Thank you for the clarification. We have removed all funding-related text from the Acknowledgments section and any other parts of the manuscript. We confirm that the Funding Statement remains unchanged and should read as follows:

“The author(s) received no specific funding for this work.”

Please proceed with updating the online submission form accordingly.

**Comment 3:** Thank you for uploading your study's underlying data set. Unfortunately, the repository you have noted in your Data Availability statement does not qualify as an acceptable data repository according to PLOS's standards.

At this time, please upload the minimal data set necessary to replicate your study's findings to a stable, public repository (such as figshare or Dryad) and provide us with the relevant URLs, DOIs, or accession numbers that may be used to access these data. For a list of recommended repositories and additional information on PLOS standards for data deposition, please see <https://journals.plos.org/plosone/s/recommended-repositories>.

**Author response:** Thank you for the suggestion. Data availability statement has been added to the revised version of the manuscript. The data used in this study are publicly available from the Demographic and Health Surveys (DHS) Program. Bangladesh Demographic and Health Survey (BDHS) datasets can be accessed upon reasonable request through the DHS Program website (<https://www.dhsprogram.com>).

**Comment 4:**  Your ethics statement should only appear in the Methods section of your manuscript. If your ethics statement is written in any section besides the Methods, please move it to the Methods section and delete it from any other section. Please ensure that your ethics statement is included in your manuscript, as the ethics statement entered into the online submission form will not be published alongside your manuscript.

**Author response:** Thank you for your instruction regarding the ethics statement. We have moved the ethics statement to the Methods section in the revised manuscript and removed it from all other sections. The ethics statement is now correctly placed in the Methods section.

**Comment 5:** Please include captions for your Supporting Information files at the end of your manuscript, and update any in-text citations to match accordingly. Please see our Supporting Information guidelines for more information: <http://journals.plos.org/plosone/s/supporting-information>.

**Author response:** Thank you for the suggestion. We have added captions for all Supporting Information files at the end of the revised manuscript. The Supporting Information now includes:

*Supplementary Table 1: Operational definition of variables*

*Supplementary Table 2: Unadjusted binary logistic regression models*

*Supplementary Table 3: Variance inflation factors (VIFs) for variables included in the multivariable models*

*Supplementary Table 4: Percentage of missing data for outcome and predictor variables*

*Supplementary Table 5: Differences between births with and without recorded birth weight based on relevant socioeconomic characteristics*

**Comment 6:** If the reviewer comments include a recommendation to cite specific previously published works, please review and evaluate these publications to determine whether they are relevant and should be cited. There is no requirement to cite these works unless the editor has indicated otherwise.

**Author response:** Thank you for the clarification. No specific publications were suggested by the reviewers. All citations included in the revised manuscript were added based on their relevance and the needs of the study, not in response to reviewer suggestions**.**

**Comment 7:** Please revise the manuscript to follow the appropriate reporting guideline. For reporting the observational study of clinical data, use the STROBE checklist ([http://www.strobe-statement.org](http://www.strobe-statement.org/)); for studies with routinely-collected data use the RECORD checklist ([http://record-statement.org](http://record-statement.org/)).

**Author response:** Thank you for this important suggestion. We have revised the manuscript in accordance with the STROBE checklist, as our study is based on observational data. The completed STROBE checklist has been provided as a supplementary file, and relevant sections of the manuscript have been updated to ensure full alignment with the reporting guidelines.

**Reviewer’s Comments**

**Reviewer #1**

**Comment:** It is an excellent piece of scientific work, with a rigorous methodology, statistical analysis and presentation of results and discussions. The results and discussions are well presented, and the conclusion is supported by the findings.

**Response:** Authors thank the reviewer for the complement.

**Comment:** Line 45: there is a grammar issue ('are significant public health issues of global concern').

**Response:** Thank you for the comment. The grammatical issue has been corrected in the revised manuscript (line 45-46).

**Comment:** On line 83, could you please provide the figures for low and child undernutrition in Bangladesh?

**Response:** Thank you for your concern. The prevalence of low birth weight was reported earlier in the Introduction (line 59). In response to the reviewer’s suggestion, figures for child undernutrition have now been added in the revised manuscript at line 84: child undernutrition (stunting 24%, wasting 11%, and underweight 22%).

**Comment:** Line 144: grammar; missing comma after 'place of delivery'.

**Response:** Thank you for the comment. The punctuation has been corrected in the revised version. (line 156).

**Comment:** From lines 260 to 269, the justification of the observed association between low birth weight (LBW) and hospital or clinic deliveries is technically unsound. Birthweight is influenced by what happens during the antenatal period, not the delivery process.

**Response:** Thank you for this important observation. We agree that birth weight is primarily influenced by factors operating during the antenatal period, rather than by the delivery process itself. We have revised the discussion accordingly and clarified the potential mediating roles of gestational age and intrauterine growth in the association between delivery-related factors and low birth weight. (line 280-296).

**Comment:** On line 326, you state that 'compared to other countries, our prevalence is lower than that found in Africa'. Africa is a continent with 54 countries. Although your statement is backed by a reference, you might want to crosscheck this.

**Response:** Thank you for your concern. The study from which the information was taken referred to the setting as Africa. However, the analysis was based on pooled data from Zambia and Kenya. We have corrected this in the revised manuscript by explicitly mentioning Zambia and Kenya at line 361.

**Comment:** In the methods section, you explain how you derived the exposure variables. I am wondering why antenatal clinic attendance was not selected, given that it is an important predictor for most of the outcome variables you selected. It would be interesting to see how this affects the outcome variables in your context.

**Response:** Thank you for the comment. Both theories driven considerations and data driven decisions were applied in selecting the variables. We agree that antenatal clinic attendance is an important predictor for most of the outcome variables. However, this variable contained a substantial proportion of missing values in our analytical sample, which could compromise the robustness of the model. Moreover, antenatal care was not significantly associated with the outcome variables in the bivariate analysis, except for preterm birth. Therefore, we couldn’t consider the variable for inferential analysis.

**Reviewer #2**

**Comment:** This manuscript uses data from the 2022 Bangladesh Demographic and Health Survey (BDHS) to estimate the prevalence of four adverse birth outcomes—stillbirth, preterm birth, low birth weight (LBW), and neonatal death—and to identify associated maternal and household characteristics using survey-weighted logistic regression. The authors also construct a composite “adverse birth outcome” (ABO) combining these four endpoints. The topic is highly relevant, and the use of a recent, nationally representative dataset is an important strength.

**Response:** Authors thank the reviewer for their valuable complement.

**Comment:** Clarity of sample selection and definition of “index pregnancy”

The description of the analytic sample is incomplete and somewhat confusing. The authors start from 73,239 pregnancy records and then exclude pregnancies before 5 years, “non-index pregnancies” (n=2,246), and terminated pregnancies/miscarriages, arriving at 10,254 pregnancies. The term “non-index pregnancy” is never defined, and readers who are not deeply familiar with BDHS data structure will not understand what the “index” pregnancy is or why non-index pregnancies are excluded.

- Please clearly define “index pregnancy” when it is first introduced (e.g. most recent birth within five years, or some other criterion) and justify why only index pregnancies are retained.
- Explicitly state how many pregnancies were excluded due to missing outcome data for each of the four outcomes (stillbirth, preterm birth, LBW, neonatal death) rather than only presenting the final denominators.
- I recommend revising or expanding the flow diagram to show, for each outcome, the initial number of pregnancies, the exclusions (with reasons), and the final analytic sample size. This is particularly important because the denominators differ substantially between outcomes (e.g. LBW vs stillbirth).

**Response:** Thank you for the comment. Non-index pregnancy was clearly defined, and the inclusion of only index pregnancies has been justified in the study sample section of the methodology in the revised version (lines 117 to 121). In the sample selection procedure, the number of cases excluded due to missing outcome data was reported, for example 7,062 cases for low birth weight. Separate analytic datasets were used for low birth weight and for other outcomes such as stillbirth. During the construction of the adverse birth outcome variable, the presence of all individual outcomes was not required; if any one outcome was present, the pregnancy was classified as having an adverse outcome. Nonetheless, the issue of missing low birth weight data has been acknowledged in the limitations section (line 483-485)

**Comment:** Handling of missing data and potential selection bias

Missing data—especially for birth weight—is a major limitation and is not handled or reported in a fully satisfactory way.

- Only 3,192 births are included in the LBW analysis, compared with 10,254 pregnancies overall, and you note that more than half of infants were not weighed at birth. This implies that the LBW models are based on a highly selected subset of the population.
- You also mention that some predictor variables have missing data and that respondents with missing data in “any of the considered outcomes” were excluded, but you do not provide a detailed breakdown of the extent and patterns of missingness.

Given this, a simple complete-case analysis can introduce serious selection bias if the probability of being weighed at birth, or of having complete covariate data, is related to socio-economic factors, access to care, or the outcomes themselves**. I strongly recommend:**

- Providing a table that presents, for each outcome and each key predictor, the number and percentage of missing observations.
- Where possible, comparing basic characteristics (e.g. maternal education, place of delivery, wealth index) between births with and without recorded birth weight, to give readers a sense of how selective the LBW subsample is.
- Considering the use of multiple imputation (e.g. MICE) for missing covariates and, if feasible, for birth weight. If this is not possible, the limitations of complete-case analysis should be discussed in more depth, particularly for the LBW models, given that more than half the potential sample is excluded.

**Response:** Thank you for your observation. Low birth weight was not recorded for a substantial proportion of cases, and the differences in socioeconomic characteristics between infants with and without recorded birth weight suggest that the LBW sample represents a non-random subsample. In line with your suggestion, we have included two tables in the Supplementary File (Supplementary Table 4 & 5) and referenced them (method section (line-127) and limitation (line-483) section) in the revised manuscript: one showing the percentage of missing data for outcome and predictor variables, and another highlighting the differences between births with and without recorded birth weight based on relevant socioeconomics. Because these data were likely missing not at random, imputation was not applied, and a complete case analysis was conducted. Following your recommendation, we have now addressed this issue in detail in the Discussion (limitations) sections of the revised manuscript (line 483-492). These revisions are given below for your convenience.

**Supplementary Table 4: The percentage of missing data for outcome and predictor variables**

| **Variable Name** | **Number of Missing value** | **Percentages** |
| --- | --- | --- |
| Low Birth Weight | 7062 | 68.9 |
| Preterm Birth | 143 | 1.4 |
| Neonatal Death | 143 | 1.4 |
| Place of delivery | 5200 | 50.7 |
| Delivery by caesarean section | 5211 | 50.8 |
| Maternal pregnancy desire | 5200 | 50.7 |
| Decision-making autonomy | 3536 | 34.5 |

**Supplementary Table 5: Differences between births with and without recorded birth weight based on relevant socioeconomics**

|  | **Birth Weight not recorded (%)** | **Birth weight recorded (%)** |
| --- | --- | --- |
| Maternal education |  |  |
| No education | 6.9 | 3.2 |
| Primary | 25.7 | 16.2 |
| Secondary | 51.6 | 54.6 |
| Higher | 15.9 | 26.1 |
| Place of delivery |  |  |
| Home | 82.5 | 7.8 |
| Hospital & Clinic | 17.5 | 92.2 |
| C-section delivery |  |  |
| No | 92.0 | 33.1 |
| Yes | 8.0 | 66.9 |
| Wealth quintile |  |  |
| Poorest | 25.0 | 13.0 |
| Poorer | 20.7 | 17.4 |
| Middle | 19.5 | 19.9 |
| Richer | 18.0 | 23.4 |
| Richest | 16.7 | 26.3 |

**Added limitation:** However, the analysis of low birth weight was restricted to births with reported birth weight, which substantially reduced the analytic sample and complete case analysis may have introduced selection bias, as 68.9 percent of infants were not weighed at birth. Births with recorded birth weight differed systematically from those without measurements. Mothers of infants with available birth weight data were significantly more likely to have secondary or higher education, belong to higher wealth quintiles, and deliver in health facilities compared with mothers whose infants were not weighed at birth (Supplementary file). These patterns indicate that the LBW analytic sample represents a more socioeconomically advantaged subset of the study population. This suspected selection bias may limit the generalizability of the LBW estimates, which should therefore be interpreted with caution.

**Comment:** Multicollinearity assessment and model specification

The methods section states that “the multicollinearity of the available variables was checked in the next stage, and highly correlated variables were deleted,” and later that a variance inflation factor (VIF) greater than 2 was used as a threshold for multicollinearity. However, in the supplementary table of VIFs, none of the variables appear to exceed this threshold, and it is not clear whether any variables were actually dropped.

- Please clarify how multicollinearity was assessed in practice: which variables were initially considered, what correlation/VIF criteria were used, and which variables (if any) were removed.
- If no variables ultimately exceeded the VIF threshold in the final models, the statement about deleting “highly correlated variables” should be revised so as not to suggest a procedure that was not actually applied.
- Beyond VIF, it would also be useful to briefly comment on conceptual overlap between some key predictors—for example, place of delivery and mode of delivery, given that the vast majority of C-sections occur in health facilities. If feasible, you might consider sensitivity analyses (e.g. with and without mode of delivery) or at least provide a conceptual justification for including both in the same models.

**Response:** We thank the reviewer for this insightful observation. Multicollinearity was assessed using variance inflation factors (VIFs), and all variables included in the models exhibited VIF values below the predefined threshold. Therefore, no variables required removal on the basis of multicollinearity. We acknowledge that the statement indicating the deletion of highly correlated variables was inaccurate, and this has been removed in the revised manuscript to avoid any misrepresentation of the analytical procedure (line-150-153). Moreover, it was further clarified in the heading of supplementary Table 3. (New title: Variance inflation factors (VIFs) for variables included in the multivariable models)

We also appreciate the reviewer’s comment regarding the potential conceptual overlap between place of delivery and mode of delivery. We recognize that, the majority of caesarean sections are performed in health facilities, which could raise concerns about redundancy between these variables. However, these variables were retained because they represent related but conceptually distinct aspects of maternal healthcare utilization. Place of delivery reflects access to and use of healthcare facilities, whereas mode of delivery captures the nature and intensity of obstetric intervention received. Importantly, facility-based delivery does not necessarily imply surgical intervention, and caesarean delivery constitutes a specific clinical pathway within facility care that may entail distinct financial, informational, and decision-making implications for women, which could independently influence perceived and experienced barriers to healthcare access.

From a statistical perspective, multicollinearity diagnostics indicated acceptable VIF values for both variables, supporting their simultaneous inclusion in the models. Additionally, sensitivity analyses excluding mode of delivery produced substantively similar results for the primary associations, indicating that the findings were robust to alternative model specifications (as an example model of adverse birth outcome without mode of delivery has been added below). These clarifications have been incorporated into the Methods sections (Predictor variables) of the manuscript to enhance transparency and interpretability (line 150-153).

**Comment:** Interpretation of caesarean section and place of delivery

The results and discussion regarding C-section and place of delivery are potentially confusing and risk being misinterpreted in a way that contradicts current global public health concerns.

From the results:

- C-section is associated with higher odds of preterm birth.
- C-section is associated with lower odds of stillbirth and neonatal death.
- The discussion emphasises “timely” C-sections and facility deliveries as beneficial, and at one point states that operative delivery “does not directly cause preterm birth.”

This interpretation requires more nuance for several reasons:

**1. C-sections define the timing of birth.**

While many C-sections are performed for legitimate medical indications, any operative delivery by definition determines the timing of birth. Particularly when C-sections are scheduled without clear indication, they can bring births forward, increasing the frequency of early-term or even preterm deliveries. Therefore, a blanket statement that operative delivery “does not directly cause preterm birth” is too categorical. This sentence should be rephrased to acknowledge that in some circumstances—especially in the context of non-medically-indicated procedures—C-sections can indeed directly lead to an earlier gestational age at delivery.

**2. Bangladesh is approaching C-section rates of ~50%.**

In Bangladesh, C-section rates are already near 50% of all births. At such levels, it is highly likely that a substantial proportion—and plausibly many or even most—C-sections are not clinically justified. In this context, interpreting an adjusted association between C-section and lower odds of stillbirth or neonatal death as evidence of “effectiveness” is problematic. Much of this association may reflect selection mechanisms (e.g. elective C-sections among relatively low-risk women, systematic differences between home and facility births) and residual confounding rather than a true protective effect of the procedure itself.

**3. Unintended long-term health burden of excessive C-sections.**

A growing body of literature documents mid- and long-term adverse consequences of unnecessary C-sections for both women and children (e.g. complications in subsequent pregnancies, abnormal placentation, uterine rupture, impacts on neonatal microbiome, and potential long-term effects on obesity, allergy, and other outcomes). Any discussion that appears to “laud” C-section performance in a country with such high rates should be carefully balanced against this broader evidence.

**I suggest:**

- Rephrasing the statement about operative delivery and preterm birth to explicitly recognise that C-sections can, in some instances—particularly when performed without strong medical indication—result in earlier deliveries and contribute to preterm or early-term births.
- Making a clear distinction between statistical predictors in your models and modifiable risk factors. The observed associations between C-section and stillbirth/neonatal death should not be interpreted as implying that further increasing C-section use would reduce these outcomes at the population level, especially in a setting where rates are already extremely high.
- Providing a more balanced discussion that acknowledges the life-saving role of medically indicated C-sections, but situates your findings within the context of C-section overuse in Bangladesh and the associated long-term health burden for mothers and children.

**Response:** We sincerely thank Reviewer for the constructive and detailed comments regarding the interpretation of cesarean section (C-section) and place of delivery in our study. In the discussion section of revised manuscript, we clarify that while obstetric complications often necessitate cesarean delivery and are themselves risk factors for preterm birth, cesarean delivery inherently determines the timing of birth ((line 328-334) and (line 365-374) and (line 393-400)). Procedures performed without strong medical indication, particularly elective or early-term C-sections, may contribute to earlier deliveries than would otherwise have occurred.

The apparent protective associations in case of stillbirth and neonatal death may reflect selection mechanisms, differences in case severity, or residual confounding rather than a causal effect. We emphasize that timely and appropriately indicated cesarean delivery may improve outcomes for selected cases, but these associations should not be interpreted as justification for expanding cesarean use at the population level. The revised paragraphs clearly state that cesarean deliveries should be performed based on clear medical indications within a high-quality emergency obstetric and neonatal care framework.

We also revised discussion of place of delivery associated with LBW and brought gestational age and intrauterine growth as the possible mediating factor. (line 289-296)

**Comment:** Role of gestational age and potential mediation

Gestational age at birth is central to the outcomes under study—preterm birth by definition, but also LBW and neonatal death—yet it is only used as a binary indicator (preterm vs term). Given accumulating evidence that early-term births (37–38 weeks) are also associated with adverse outcomes, and that scheduled C-sections in particular can shift the gestational age distribution towards earlier births, this seems like a missed opportunity.

- If the data permit, I encourage you to explore gestational age with more granularity, for example by distinguishing preterm, early-term, and full-term births or by modelling gestational age as a continuous variable.
- From a causal perspective, gestational age is also a plausible mediator between mode of delivery and LBW or neonatal death. Even if a full formal mediation analysis is not feasible, it would be helpful to acknowledge this pathway explicitly and, if possible, conduct exploratory analyses (e.g. models with and without gestational age) to see how associations with mode of delivery change.
- In addition, in the discussion of LBW at home vs facility births, the role of gestational age and fetal growth could be clarified. LBW is primarily determined by intrauterine growth and gestational age, which are influenced by maternal health, nutrition and antenatal care over many months, rather than by intrapartum care alone. Place of delivery is likely acting as a proxy for broader socio-economic and care trajectories, and this distinction should be made clear.

**Response:** Thank you for your comment. We used the BDHS variable P21, which measures duration of pregnancy; however, this variable is reported in weeks or months rather than at the day level. Given this limitation, and in line with our study objectives, gestational age was operationalized as a binary variable, as preterm birth was one of the primary outcomes of interest. We acknowledge that this approach does not allow examination of finer gradations, such as early-term (37–38 weeks) versus full-term births.

From a causal perspective, gestational age may plausibly lie on the pathway between mode of delivery and outcomes such as low birth weight or neonatal death. Although a formal mediation analysis was not conducted, we recognize the importance of interpreting the associations between mode of delivery and these outcomes in light of this potential mechanism. Following your suggestion, we have revised the discussion to more explicitly describe these pathways, including the roles of gestational age and intrauterine growth, in the sections on both low birth weight and neonatal death (line 289-296) and (line 396-398).

**Comment:** Twin births and preterm birth: implausible protective association

In the preterm birth models, twin births appear to have substantially lower odds of preterm birth than singletons (i.e. twins seem “protective” against prematurity), which directly contradicts a large body of obstetric literature. The discussion offers several ad hoc explanations (e.g. misclassification, more monitoring of twin pregnancies), but given the magnitude and direction of the effect, this pattern is suspicious and may reflect coding or modelling issues.

- Please re-check the coding of the twin variable, the coding of the preterm variable, and the reference category used in the regression models.
- Verify that multiple births have been correctly identified in the BDHS data and that gestational age categorisation is correct.
- If, after these checks, the result persists, it should be presented with much more caution, clearly framed as likely driven by measurement or classification problems rather than a true protective effect of twinning.

**Response:** We thank the reviewer for this important observation. We re-verified the coding of the multiple birth variable, the definition of preterm birth (<37 completed weeks), and the reference categories used in the regression models, following the BDHS recode manual. Multiple births and gestational age were correctly identified. In unadjusted analyses, twin births were associated with approximately two-fold higher odds of preterm birth, consistent with the established obstetric literature. However, in the fully adjusted model, twin births showed a statistically significant inverse association with preterm birth (AOR 0.13, 95% CI 0.02–0.98). Given the very small number of twin births (n = 15) and the wide confidence interval, we consider this estimate to be unstable. Moreover, the reversal of the association after adjustment likely reflects over-adjustment and residual confounding, particularly due to inclusion of delivery-related variables such as place of delivery and caesarean section, which are downstream consequences of high-risk pregnancies and are strongly associated with both twin gestation and preterm birth. Given the strong prior evidence that twin pregnancies carry substantially higher risk of prematurity, we do not interpret the adjusted inverse association as a true protective effect. We have revised the manuscript to clearly state that this pattern is likely driven by residual confounding, over-adjustment, or measurement and classification limitations of gestational age in DHS data, particularly for multiple births (line 337-339 & 345). Accordingly, we have substantially tempered the interpretation of this result and now discuss it primarily as a methodological limitation rather than a substantive finding in the discussion section.

**Comment:** Composite “adverse birth outcome” definition

The composite “adverse birth outcome” that combines stillbirth, preterm birth, LBW and neonatal death is not fully justified conceptually. These outcomes have distinct etiologies, and some are conditioned on others (e.g. neonatal death is conditional on live birth).

- Please provide a clearer rationale for constructing this composite outcome. What clinical or public health construct is it meant to represent?
- It would also be helpful to report how many infants experienced each component and how much the composite is driven by the more common outcomes (e.g. LBW) versus the rarer endpoints (stillbirth, neonatal death).
- Given that you already present separate models for each individual outcome, the added value of the composite should be made explicit, and its limitations discussed.

**Response:** Thank you for this important and constructive comment. We agree that stillbirth, preterm birth, low birth weight, and neonatal death have distinct etiologies and that some outcomes are conditionally related. Our intention in constructing a composite “adverse birth outcome” was not to imply a single biological pathway, but rather to capture an overarching perinatal vulnerability construct reflecting serious adverse outcomes across the continuum from late pregnancy through the early neonatal period. As similar types of approaches were taken in several literatures [Determinants of adverse birth outcome in Sub-Saharan Africa: analysis of recent demographic and health surveys – PMC (doi: 10.1186/s12889-021-11113-z) ; Adverse birth outcomes and its associated factors among women who delivered in North Wollo zone, northeast Ethiopia: a facility based cross-sectional study – PMC (doi: 10.1186/s13104-019-4387-9)].

However, following your suggestion, we are including these references with objectives for composition in the method section’s outcome variable subsection [This composite outcome was intended to capture overall perinatal vulnerability across the late pregnancy and early neonatal period rather than to represent a single etiological process.] in the revised version (line140-142). We have also revised the description of Figure 2 to clarify that the composite indicator is largely driven by common outcomes such as low birth weight and preterm birth, which may obscure associations with rarer outcomes (line 211-2012). To ensure the robustness of our findings, we also examined associations for each individual outcome separately. Nevertheless, this limitation has been explicitly mentioned in the revised version following your suggestion [Another issue is that the composite adverse birth outcome includes events with distinct etiologies and conditional relationships and is largely driven by common outcomes, such as low birth weight and preterm birth, which may obscure associations with rarer outcomes. To ensure robustness of conclusion, we therefore examined each outcome separately] (line 494-498)

**Comment:** Terminology in regression models

The manuscript repeatedly refers to “multivariate binary logistic regression.” Since the models involve multiple predictors and a single binary outcome, the standard term in epidemiology is “multivariable logistic regression.” I suggest using “multivariable” consistently throughout.

**Response:** Thank you for your nice observation. We changed multivariate to multivariable in the revised version (170-171).

**Comment:** Definition and referencing of the wealth index

The wealth index is a key covariate. Although it is standard in DHS surveys, a brief description (e.g. asset-based index constructed using principal components analysis) and/or an explicit reference to DHS documentation would help readers unfamiliar with this measure.

**Response:** Thank you. Following your suggestion we included detailed about wealth index with appropriate reference in the revised version. [Household socioeconomic status was measured using the wealth index, a composite indicator of cumulative living standards derived from household asset ownership through principal component analysis]. (Lines 161-163). Also details information can be available from supplementary documents.

**Comment:** Clarification of n (%) in tables

In Tables 2–4, the “n (%)” entries are smaller than the overall sample size and the percentages do not sum to 100% across categories. It appears that “n (%)” refers to the number and percentage of births experiencing the outcome within each covariate category. If this is the case, please state it clearly in a table footnote to avoid confusion.

**Response:** Thank you for your observation. Following your suggestion note has been added to each table to avoid confusion in the revised version. (Below Table 2-4)

**Comment:** Abstract conclusions

The conclusions in the abstract are somewhat generic and largely restate well-known facts. I suggest revising the abstract conclusion to highlight a small number of specific findings (e.g. the magnitude of LBW prevalence, the pattern of associations with wealth index and women’s decision-making autonomy, or the high C-section rate) and to offer 1–2 concrete, context-specific policy implications for Bangladesh.

**Response:** Thank you for your suggestion. We have revised the conclusion section highlighting the key findings and result-oriented policy implications. Please see the revised conclusion section as “One in seven births in Bangladesh involved at least one adverse outcome, with LBW being the most prevalent. Socioeconomic disadvantage and limited women’s decision-making autonomy were consistently associated with adverse outcomes. Policies should prioritize equity-oriented maternal care, strengthen women’s autonomy, and ensure appropriate use of obstetric interventions.” In the lines 34-38 in the revised manuscript.

**Comment:** Language and typographical issues

The manuscript would benefit from careful language editing by a fluent English speaker. There are multiple minor typographical and grammatical errors (e.g. “servey”, “isuue”, “one women”, etc.) and some sentences in the Discussion and Limitations sections are awkwardly phrased.

**Response:** Thank you for your comment. Typographical and grammatical errors have been corrected throughout the revised manuscript.

**Comment:** Overall, this is a potentially valuable analysis of BDHS 2022 data on adverse birth outcomes, but the issues outlined above—especially around sample definition, missing data, and the interpretation of C-sections and gestational age—need to be addressed for the paper to make a robust and balanced contribution.

**Response:** We appreciate the valuable feedback from the reviewer. We have tried to address the issues raised by the reviewer and revised the manuscript following the suggestions. Please see the changes in the revised manuscript. Hopefully, the revised version of the manuscript presents a methodologically sound findings with proper interpretation.
