# Supplementary material for: Prevalence and risk factors of adverse birth outcomes in Bangladesh: Insight from a nationwide survey
Source: PLoS One. 2026 Jun 17;21(6):e0351676. doi: 10.1371/journal.pone.0351676 (PMC13274831; doi:10.1371/journal.pone.0351676)
Supplement: Attachment — Submitted filename: Response to Reviewers.docx [file pone.0351676.s003.docx]

Reviewer’s Comments

Reviewer #1

Comment: Line 88, It would be better to say significantly reduce adverse birth outcomes, as logically, it is not possible to halt adverse birth outcomes and their consequences in a country.

Response: Thank you for your comment. We have changed halt to significantly reduce based on your advice in the revised manuscript (line 88).

Comment: Line 173, preterm pregnancy, I would suggest consistency in the use of terms throughout the paper, other sections of the paper use preterm birth.

Response: Thank you for your valuable comment. We have corrected “preterm pregnancy” to “preterm birth” (line 173). Additionally, “preterm labor” has been revised to “preterm birth” in line 380 of the revised manuscript. We have carefully reviewed the entire document to ensure consistency, and no discrepancies remain; the terms “preterm birth” and “preterm delivery” are now used appropriately according to the context.

Comment: It would be worthwhile stating the discrepancy in your findings regarding the association between twin pregnancies, low birth weight and preterm delivery together. While some sections of your discussion mention them together as possible risk factors for adverse outcomes, your findings seem to contradict this.

Response: We thank the reviewer for this valuable comment and for highlighting the need for clearer interpretation. We acknowledge the apparent inconsistency between the established role of twin pregnancies as risk factors for adverse outcomes and the inverse association observed with preterm birth in our findings.

In response, we have revised the preterm birth section of the Discussion (lines 345–348) to explicitly address this discrepancy and to clarify that the observed inverse association contrasts with existing evidence and should be interpreted with caution.

Additionally, we have revised the wording in the adverse birth outcome section by explicitly referring to the outcome as a composite adverse birth outcome (line 439). This clarification ensures that the interpretation reflects the combined nature of the indicator, rather than implying a uniform effect across all individual outcomes, thereby reducing potential confusion for readers.

Reviewer #2

Comment: Authors adequately addressed prior concerns; methods and interpretation now sound. Recommend acceptance; only minor language/typo edits remain.

Response: Thank you for the comment. The manuscript has been revised for the typo and other language correction.
