# Supplementary material for: Prevalence and risk factors of adverse birth outcomes in Bangladesh: Insight from a nationwide survey
Source: PLoS One. 2026 Jun 17;21(6):e0351676. doi: 10.1371/journal.pone.0351676 (PMC13274831; doi:10.1371/journal.pone.0351676)
Supplement: Attachment — Submitted filename: Response to Journal Requirements_R3.docx [file pone.0351676.s004.docx]

**Response to Journal Requirements:**

1. If the reviewer comments include a recommendation to cite specific previously published works, please review and evaluate these publications to determine whether they are relevant and should be cited. There is no requirement to cite these works unless the editor has indicated otherwise.

**Response:** We thank the editor for this note. We carefully reviewed all reviewer comments and confirm that no recommendations were made to cite specific previously published works.

2. Please review your reference list to ensure that it is complete and correct. If you have cited papers that have been retracted, please include the rationale for doing so in the manuscript text, or remove these references and replace them with relevant current references. Any changes to the reference list should be mentioned in the rebuttal letter that accompanies your revised manuscript. If you need to cite a retracted article, indicate the article’s retracted status in the References list and also include a citation and full reference for the retraction notice.

**Response:** We carefully reviewed the entire reference list to ensure that all references are complete, accurate, and up to date. We also checked for any retracted publications and confirm that no retracted articles have been cited in the manuscript.

**Response to Additional Editor Comments:**

1. The authors have addressed many of the reviewer comments; however, some issues remain unresolved. In particular, discrepancies persist in the use of terminology related to “preterm birth” between the main text and figures. Please ensure consistent usage throughout the manuscript.

**Response:** Thank you for this important observation. We have carefully revised the manuscript to ensure consistent use of the term “preterm birth” throughout the text and figures. In addition, the term “preterm delivery” was replaced with “preterm birth” to maintain terminological consistency across the manuscript.

2. In addition, please standardize the capitalization format (sentence case vs. title case) across all figure and table titles in accordance with the journal’s guidelines.

**Response:** Thank you for this valuable suggestion. We have carefully revised all figure and table titles throughout the manuscript and standardized their capitalization format in accordance with the journal’s guidelines. Specifically, sentence case formatting has now been applied across all figure and table titles.
