# Supplementary material for: Prevalence and risk factors of adverse birth outcomes in Bangladesh: Insight from a nationwide survey
Source: PLoS One. 2026 Jun 17;21(6):e0351676. doi: 10.1371/journal.pone.0351676 (PMC13274831; doi:10.1371/journal.pone.0351676)
Supplement: Peer review history

# Peer review: Decision Letter 0

**DOI:** 10.1371/journal.pone.0351676.r001

**Authors:** Md. Obaidur Rahman

29 Dec 2025

PONE-D-25-43758

Prevalence and risk factors of adverse birth outcomes in Bangladesh: Insight from a nationwide survey

PLOS One

Dear Dr. Islam,

Thank you for submitting your manuscript to PLOS ONE. After careful consideration, we feel that it has merit but does not fully meet PLOS ONE’s publication criteria as it currently stands. Therefore, we invite you to submit a revised version of the manuscript that addresses the points raised during the review process.

Please submit your revised manuscript by Feb 12 2026 11:59PM. If you will need more time than this to complete your revisions, please reply to this message or contact the journal office at plosone@plos.org. When you're ready to submit your revision, log on to https://www.editorialmanager.com/pone/ and select the 'Submissions Needing Revision' folder to locate your manuscript file.

Please include the following items when submitting your revised manuscript:

- A letter that responds to each point raised by the academic editor and reviewer(s). You should upload this letter as a separate file labeled 'Response to Reviewers'.
- A marked-up copy of your manuscript that highlights changes made to the original version. You should upload this as a separate file labeled 'Revised Manuscript with Track Changes'.
- An unmarked version of your revised paper without tracked changes. You should upload this as a separate file labeled 'Manuscript'.

If you would like to make changes to your financial disclosure, please include your updated statement in your cover letter. Guidelines for resubmitting your figure files are available below the reviewer comments at the end of this letter.

If applicable, we recommend that you deposit your laboratory protocols in protocols.io to enhance the reproducibility of your results. Protocols.io assigns your protocol its own identifier (DOI) so that it can be cited independently in the future. For instructions see: https://journals.plos.org/plosone/s/submission-guidelines#loc-laboratory-protocols. Additionally, PLOS ONE offers an option for publishing peer-reviewed Lab Protocol articles, which describe protocols hosted on protocols.io. Read more information on sharing protocols at https://plos.org/protocols?utm_medium=editorial-email&utm_source=authorletters&utm_campaign=protocols.

We look forward to receiving your revised manuscript.

Kind regards,

Md. Obaidur Rahman, Ph.D.

Academic Editor

PLOS One

Journal Requirements:

When submitting your revision, we need you to address these additional requirements.

1. Please ensure that your manuscript meets PLOS ONE's style requirements, including those for file naming. The PLOS ONE style templates can be found at

https://journals.plos.org/plosone/s/file?id=wjVg/PLOSOne_formatting_sample_main_body.pdf and

https://journals.plos.org/plosone/s/file?id=ba62/PLOSOne_formatting_sample_title_authors_affiliations.pdf

2. Thank you for stating the following in the Acknowledgments Section of your manuscript:

“This research received no specific grant from any funding agency in the public, commercial or not-for-profit sectors.”

We note that you have provided funding information that is not currently declared in your Funding Statement. However, funding information should not appear in the Acknowledgments section or other areas of your manuscript. We will only publish funding information present in the Funding Statement section of the online submission form.

Please remove any funding-related text from the manuscript and let us know how you would like to update your Funding Statement. Currently, your Funding Statement reads as follows:

“The author(s) received no specific funding for this work.”

Please include your amended statements within your cover letter; we will change the online submission form on your behalf.

3. Thank you for uploading your study's underlying data set. Unfortunately, the repository you have noted in your Data Availability statement does not qualify as an acceptable data repository according to PLOS's standards.

At this time, please upload the minimal data set necessary to replicate your study's findings to a stable, public repository (such as figshare or Dryad) and provide us with the relevant URLs, DOIs, or accession numbers that may be used to access these data. For a list of recommended repositories and additional information on PLOS standards for data deposition, please see https://journals.plos.org/plosone/s/recommended-repositories.

4. Your ethics statement should only appear in the Methods section of your manuscript. If your ethics statement is written in any section besides the Methods, please move it to the Methods section and delete it from any other section. Please ensure that your ethics statement is included in your manuscript, as the ethics statement entered into the online submission form will not be published alongside your manuscript.

5. Please include captions for your Supporting Information files at the end of your manuscript, and update any in-text citations to match accordingly. Please see our Supporting Information guidelines for more information: http://journals.plos.org/plosone/s/supporting-information.

6. If the reviewer comments include a recommendation to cite specific previously published works, please review and evaluate these publications to determine whether they are relevant and should be cited. There is no requirement to cite these works unless the editor has indicated otherwise.

Additional Editor Comments:

Please revise the manuscript to follow the appropriate reporting guideline. For reporting the observational study of clinical data , use the STROBE checklist (http://www.strobe-statement.org); for studies with routinely-collected data use the RECORD checklist (http://record-statement.org).

[Note: HTML markup is below. Please do not edit.]

Reviewers' comments:

Reviewer's Responses to Questions

**Comments to the Author**

1. Is the manuscript technically sound, and do the data support the conclusions?

The manuscript must describe a technically sound piece of scientific research with data that supports the conclusions. Experiments must have been conducted rigorously, with appropriate controls, replication, and sample sizes. The conclusions must be drawn appropriately based on the data presented.

Reviewer #1: Partly

Reviewer #2: Partly

**********

2. Has the statistical analysis been performed appropriately and rigorously?

Reviewer #1: Yes

Reviewer #2: Yes

**********

3. Have the authors made all data underlying the findings in their manuscript fully available?

The PLOS Data policy requires authors to make all data underlying the findings described in their manuscript fully available without restriction, with rare exception (please refer to the Data Availability Statement in the manuscript PDF file). The data should be provided as part of the manuscript or its supporting information, or deposited to a public repository. For example, in addition to summary statistics, the data points behind means, medians and variance measures should be available. If there are restrictions on publicly sharing data—e.g. participant privacy or use of data from a third party—those must be specified.

Reviewer #1: Yes

Reviewer #2: Yes

**********

4. Is the manuscript presented in an intelligible fashion and written in standard English?

PLOS ONE does not copyedit accepted manuscripts, so the language in submitted articles must be clear, correct, and unambiguous. Any typographical or grammatical errors should be corrected at revision, so please note any specific errors here.

Reviewer #1: Yes

Reviewer #2: Yes

**********

5. Review Comments to the Author

Please use the space provided to explain your answers to the questions above. You may also include additional comments for the author, including concerns about dual publication, research ethics, or publication ethics. (Please upload your review as an attachment if it exceeds 20,000 characters)

Reviewer #1: It is an excellent piece of scientific work, with a rigorous methodology, statistical analysis and presentation of results and discussions. The results and discussions are well presented, and the conclusion is supported by the findings. However, I have made some remarks to improve the paper further.

1. Line 45: there is a grammar issue ('are significant public health issues of global concern').

2. On line 83, could you please provide the figures for low and child undernutrition in Bangladesh?

3. Line 144: grammar; missing comma after 'place of delivery'.

4. From lines 260 to 269, the justification of the observed association between low birth weight (LBW) and hospital or clinic deliveries is technically unsound. Birthweight is influenced by what happens during the antenatal period, not the delivery process.

5. On line 326, you state that 'compared to other countries, our prevalence is lower than that found in Africa'. Africa is a continent with 54 countries. Although your statement is backed by a reference, you might want to cross-check this.

In the methods section, you explain how you derived the exposure variables. I am wondering why antenatal clinic attendance was not selected, given that it is an important predictor for most of the outcome variables you selected. It would be interesting to see how this affects the outcome variables in your context.

Reviewer #2: This manuscript uses data from the 2022 Bangladesh Demographic and Health Survey (BDHS) to estimate the prevalence of four adverse birth outcomes—stillbirth, preterm birth, low birth weight (LBW), and neonatal death—and to identify associated maternal and household characteristics using survey-weighted logistic regression. The authors also construct a composite “adverse birth outcome” (ABO) combining these four endpoints.

The topic is highly relevant, and the use of a recent, nationally representative dataset is an important strength. However, there are significant concerns regarding: (1) the clarity of sample definition, (2) the handling and reporting of missing data (particularly for birth weight), (3) the assessment and interpretation of multicollinearity, and (4) the conceptual and causal interpretation of mode of delivery, gestational age, and place of birth, especially in the context of very high caesarean section (C-section) rates in Bangladesh. Substantial revisions are required before the manuscript can be considered further.

Major comments

1. Clarity of sample selection and definition of “index pregnancy”

The description of the analytic sample is incomplete and somewhat confusing. The authors start from 73,239 pregnancy records and then exclude pregnancies before 5 years, “non-index pregnancies” (n=2,246), and terminated pregnancies/miscarriages, arriving at 10,254 pregnancies. The term “non-index pregnancy” is never defined, and readers who are not deeply familiar with BDHS data structure will not understand what the “index” pregnancy is or why non-index pregnancies are excluded.

• Please clearly define “index pregnancy” when it is first introduced (e.g. most recent birth within five years, or some other criterion) and justify why only index pregnancies are retained.

• Explicitly state how many pregnancies were excluded due to missing outcome data for each of the four outcomes (stillbirth, preterm birth, LBW, neonatal death) rather than only presenting the final denominators.

• I recommend revising or expanding the flow diagram to show, for each outcome, the initial number of pregnancies, the exclusions (with reasons), and the final analytic sample size. This is particularly important because the denominators differ substantially between outcomes (e.g. LBW vs stillbirth).

2. Handling of missing data and potential selection bias

Missing data—especially for birth weight—is a major limitation and is not handled or reported in a fully satisfactory way.

• Only 3,192 births are included in the LBW analysis, compared with 10,254 pregnancies overall, and you note that more than half of infants were not weighed at birth. This implies that the LBW models are based on a highly selected subset of the population.

• You also mention that some predictor variables have missing data and that respondents with missing data in “any of the considered outcomes” were excluded, but you do not provide a detailed breakdown of the extent and patterns of missingness.

Given this, a simple complete-case analysis can introduce serious selection bias if the probability of being weighed at birth, or of having complete covariate data, is related to socio-economic factors, access to care, or the outcomes themselves.

I strongly recommend:

• Providing a table that presents, for each outcome and each key predictor, the number and percentage of missing observations.

• Where possible, comparing basic characteristics (e.g. maternal education, place of delivery, wealth index) between births with and without recorded birth weight, to give readers a sense of how selective the LBW subsample is.

• Considering the use of multiple imputation (e.g. MICE) for missing covariates and, if feasible, for birth weight. If this is not possible, the limitations of complete-case analysis should be discussed in more depth, particularly for the LBW models, given that more than half the potential sample is excluded.

3. Multicollinearity assessment and model specification

The methods section states that “the multicollinearity of the available variables was checked in the next stage, and highly correlated variables were deleted,” and later that a variance inflation factor (VIF) greater than 2 was used as a threshold for multicollinearity. However, in the supplementary table of VIFs, none of the variables appear to exceed this threshold, and it is not clear whether any variables were actually dropped.

• Please clarify how multicollinearity was assessed in practice: which variables were initially considered, what correlation/VIF criteria were used, and which variables (if any) were removed.

• If no variables ultimately exceeded the VIF threshold in the final models, the statement about deleting “highly correlated variables” should be revised so as not to suggest a procedure that was not actually applied.

• Beyond VIF, it would also be useful to briefly comment on conceptual overlap between some key predictors—for example, place of delivery and mode of delivery, given that the vast majority of C-sections occur in health facilities. If feasible, you might consider sensitivity analyses (e.g. with and without mode of delivery) or at least provide a conceptual justification for including both in the same models.

4. Interpretation of caesarean section and place of delivery

The results and discussion regarding C-section and place of delivery are potentially confusing and risk being misinterpreted in a way that contradicts current global public health concerns.

From the results:

• C-section is associated with higher odds of preterm birth.

• C-section is associated with lower odds of stillbirth and neonatal death.

• The discussion emphasises “timely” C-sections and facility deliveries as beneficial, and at one point states that operative delivery “does not directly cause preterm birth.”

This interpretation requires more nuance for several reasons:

1. C-sections define the timing of birth.

While many C-sections are performed for legitimate medical indications, any operative delivery by definition determines the timing of birth. Particularly when C-sections are scheduled without clear indication, they can bring births forward, increasing the frequency of early-term or even preterm deliveries. Therefore, a blanket statement that operative delivery “does not directly cause preterm birth” is too categorical. This sentence should be rephrased to acknowledge that in some circumstances—especially in the context of non-medically-indicated procedures—C-sections can indeed directly lead to an earlier gestational age at delivery.

2. Bangladesh is approaching C-section rates of ~50%.

In Bangladesh, C-section rates are already near 50% of all births. At such levels, it is highly likely that a substantial proportion—and plausibly many or even most—C-sections are not clinically justified. In this context, interpreting an adjusted association between C-section and lower odds of stillbirth or neonatal death as evidence of “effectiveness” is problematic. Much of this association may reflect selection mechanisms (e.g. elective C-sections among relatively low-risk women, systematic differences between home and facility births) and residual confounding rather than a true protective effect of the procedure itself.

3. Unintended long-term health burden of excessive C-sections.

A growing body of literature documents mid- and long-term adverse consequences of unnecessary C-sections for both women and children (e.g. complications in subsequent pregnancies, abnormal placentation, uterine rupture, impacts on neonatal microbiome, and potential long-term effects on obesity, allergy, and other outcomes). Any discussion that appears to “laud” C-section performance in a country with such high rates should be carefully balanced against this broader evidence.

I suggest:

• Rephrasing the statement about operative delivery and preterm birth to explicitly recognise that C-sections can, in some instances—particularly when performed without strong medical indication—result in earlier deliveries and contribute to preterm or early-term births.

• Making a clear distinction between statistical predictors in your models and modifiable risk factors. The observed associations between C-section and stillbirth/neonatal death should not be interpreted as implying that further increasing C-section use would reduce these outcomes at the population level, especially in a setting where rates are already extremely high.

• Providing a more balanced discussion that acknowledges the life-saving role of medically indicated C-sections, but situates your findings within the context of C-section overuse in Bangladesh and the associated long-term health burden for mothers and children.

5. Role of gestational age and potential mediation

Gestational age at birth is central to the outcomes under study—preterm birth by definition, but also LBW and neonatal death—yet it is only used as a binary indicator (preterm vs term). Given accumulating evidence that early-term births (37–38 weeks) are also associated with adverse outcomes, and that scheduled C-sections in particular can shift the gestational age distribution towards earlier births, this seems like a missed opportunity.

• If the data permit, I encourage you to explore gestational age with more granularity, for example by distinguishing preterm, early-term, and full-term births or by modelling gestational age as a continuous variable.

• From a causal perspective, gestational age is also a plausible mediator between mode of delivery and LBW or neonatal death. Even if a full formal mediation analysis is not feasible, it would be helpful to acknowledge this pathway explicitly and, if possible, conduct exploratory analyses (e.g. models with and without gestational age) to see how associations with mode of delivery change.

• In addition, in the discussion of LBW at home vs facility births, the role of gestational age and fetal growth could be clarified. LBW is primarily determined by intrauterine growth and gestational age, which are influenced by maternal health, nutrition and antenatal care over many months, rather than by intrapartum care alone. Place of delivery is likely acting as a proxy for broader socio-economic and care trajectories, and this distinction should be made clear.

6. Twin births and preterm birth: implausible protective association

In the preterm birth models, twin births appear to have substantially lower odds of preterm birth than singletons (i.e. twins seem “protective” against prematurity), which directly contradicts a large body of obstetric literature. The discussion offers several ad hoc explanations (e.g. misclassification, more monitoring of twin pregnancies), but given the magnitude and direction of the effect, this pattern is suspicious and may reflect coding or modelling issues.

• Please re-check the coding of the twin variable, the coding of the preterm variable, and the reference category used in the regression models.

• Verify that multiple births have been correctly identified in the BDHS data and that gestational age categorisation is correct.

• If, after these checks, the result persists, it should be presented with much more caution, clearly framed as likely driven by measurement or classification problems rather than a true protective effect of twinning.

7. Composite “adverse birth outcome” definition

The composite “adverse birth outcome” that combines stillbirth, preterm birth, LBW and neonatal death is not fully justified conceptually. These outcomes have distinct etiologies, and some are conditioned on others (e.g. neonatal death is conditional on live birth).

• Please provide a clearer rationale for constructing this composite outcome. What clinical or public health construct is it meant to represent?

• It would also be helpful to report how many infants experienced each component and how much the composite is driven by the more common outcomes (e.g. LBW) versus the rarer endpoints (stillbirth, neonatal death).

• Given that you already present separate models for each individual outcome, the added value of the composite should be made explicit, and its limitations discussed.

Minor comments

1. Terminology in regression models

o The manuscript repeatedly refers to “multivariate binary logistic regression.” Since the models involve multiple predictors and a single binary outcome, the standard term in epidemiology is “multivariable logistic regression.” I suggest using “multivariable” consistently throughout.

2. Definition and referencing of the wealth index

o The wealth index is a key covariate. Although it is standard in DHS surveys, a brief description (e.g. asset-based index constructed using principal components analysis) and/or an explicit reference to DHS documentation would help readers unfamiliar with this measure.

3. Clarification of n (%) in tables

o In Tables 2–4, the “n (%)” entries are smaller than the overall sample size and the percentages do not sum to 100% across categories. It appears that “n (%)” refers to the number and percentage of births experiencing the outcome within each covariate category. If this is the case, please state it clearly in a table footnote to avoid confusion.

4. Abstract conclusions

o The conclusions in the abstract are somewhat generic and largely restate well-known facts. I suggest revising the abstract conclusion to highlight a small number of specific findings (e.g. the magnitude of LBW prevalence, the pattern of associations with wealth index and women’s decision-making autonomy, or the high C-section rate) and to offer 1–2 concrete, context-specific policy implications for Bangladesh.

5. Language and typographical issues

o The manuscript would benefit from careful language editing by a fluent English speaker. There are multiple minor typographical and grammatical errors (e.g. “servey”, “isuue”, “one women”, etc.) and some sentences in the Discussion and Limitations sections are awkwardly phrased.

Overall, this is a potentially valuable analysis of BDHS 2022 data on adverse birth outcomes, but the issues outlined above—especially around sample definition, missing data, and the interpretation of C-sections and gestational age—need to be addressed for the paper to make a robust and balanced contribution.

**********

6. PLOS authors have the option to publish the peer review history of their article (what does this mean?). If published, this will include your full peer review and any attached files.

If you choose “no”, your identity will remain anonymous but your review may still be made public.

**Do you want your identity to be public for this peer review?**   For information about this choice, including consent withdrawal, please see our Privacy Policy.

Reviewer #1: **Yes:**   CHANDINI ALIYOU MOUSTAPHA

Reviewer #2: No

**********

[NOTE: If reviewer comments were submitted as an attachment file, they will be attached to this email and accessible via the submission site. Please log into your account, locate the manuscript record, and check for the action link "View Attachments". If this link does not appear, there are no attachment files.]

To ensure your figures meet our technical requirements, please review our figure guidelines: https://journals.plos.org/plosone/s/figures

You may also use PLOS’s free figure tool, NAAS, to help you prepare publication quality figures: https://journals.plos.org/plosone/s/figures#loc-tools-for-figure-preparation.

NAAS will assess whether your figures meet our technical requirements by comparing each figure against our figure specifications.

# Author comment: Author response to Decision Letter 1

**DOI:** 10.1371/journal.pone.0351676.r002

2 Feb 2026

Author's responses to the editor's and reviewers' comments have been provided in a separate file.

Attachment Submitted filename: renamed_08b74.docx

# Peer review: Decision Letter 1

**DOI:** 10.1371/journal.pone.0351676.r003

**Authors:** Md. Obaidur Rahman

6 Mar 2026

PONE-D-25-43758R1

Prevalence and risk factors of adverse birth outcomes in Bangladesh: Insight from a nationwide survey

PLOS One

Dear Dr. Islam,

Thank you for submitting your manuscript to PLOS ONE. After careful consideration, we feel that it has merit but does not fully meet PLOS ONE’s publication criteria as it currently stands. Therefore, we invite you to submit a revised version of the manuscript that addresses the points raised during the review process.

Please submit your revised manuscript by Apr 20 2026 11:59PM. If you will need more time than this to complete your revisions, please reply to this message or contact the journal office at plosone@plos.org. When you're ready to submit your revision, log on to https://www.editorialmanager.com/pone/ and select the 'Submissions Needing Revision' folder to locate your manuscript file.

Please include the following items when submitting your revised manuscript:

- A letter that responds to each point raised by the academic editor and reviewer(s). You should upload this letter as a separate file labeled 'Response to Reviewers'.
- A marked-up copy of your manuscript that highlights changes made to the original version. You should upload this as a separate file labeled 'Revised Manuscript with Track Changes'.
- An unmarked version of your revised paper without tracked changes. You should upload this as a separate file labeled 'Manuscript'.

-->If you would like to make changes to your financial disclosure, please include your updated statement in your cover letter. Guidelines for resubmitting your figure files are available below the reviewer comments at the end of this letter.

If applicable, we recommend that you deposit your laboratory protocols in protocols.io to enhance the reproducibility of your results. Protocols.io assigns your protocol its own identifier (DOI) so that it can be cited independently in the future. For instructions see: https://journals.plos.org/plosone/s/submission-guidelines#loc-laboratory-protocols. Additionally, PLOS ONE offers an option for publishing peer-reviewed Lab Protocol articles, which describe protocols hosted on protocols.io. Read more information on sharing protocols at https://plos.org/protocols?utm_medium=editorial-email&utm_source=authorletters&utm_campaign=protocols.

We look forward to receiving your revised manuscript.

Kind regards,

Md. Obaidur Rahman, Ph.D.

Academic Editor

PLOS One

Journal Requirements:

1. If the reviewer comments include a recommendation to cite specific previously published works, please review and evaluate these publications to determine whether they are relevant and should be cited. There is no requirement to cite these works unless the editor has indicated otherwise.

2. Please review your reference list to ensure that it is complete and correct. If you have cited papers that have been retracted, please include the rationale for doing so in the manuscript text, or remove these references and replace them with relevant current references. Any changes to the reference list should be mentioned in the rebuttal letter that accompanies your revised manuscript. If you need to cite a retracted article, indicate the article’s retracted status in the References list and also include a citation and full reference for the retraction notice.

[Note: HTML markup is below. Please do not edit.]

Reviewers' comments:

Reviewer's Responses to Questions

**Comments to the Author**

1. If the authors have adequately addressed your comments raised in a previous round of review and you feel that this manuscript is now acceptable for publication, you may indicate that here to bypass the “Comments to the Author” section, enter your conflict of interest statement in the “Confidential to Editor” section, and submit your "Accept" recommendation.

Reviewer #1: All comments have been addressed

Reviewer #2: All comments have been addressed

**********

2. Is the manuscript technically sound, and do the data support the conclusions?

The manuscript must describe a technically sound piece of scientific research with data that supports the conclusions. Experiments must have been conducted rigorously, with appropriate controls, replication, and sample sizes. The conclusions must be drawn appropriately based on the data presented.

Reviewer #1: Yes

Reviewer #2: Yes

**********

3. Has the statistical analysis been performed appropriately and rigorously?

Reviewer #1: Yes

Reviewer #2: Yes

**********

4. Have the authors made all data underlying the findings in their manuscript fully available?

The PLOS Data policy requires authors to make all data underlying the findings described in their manuscript fully available without restriction, with rare exception (please refer to the Data Availability Statement in the manuscript PDF file). The data should be provided as part of the manuscript or its supporting information, or deposited to a public repository. For example, in addition to summary statistics, the data points behind means, medians and variance measures should be available. If there are restrictions on publicly sharing data—e.g. participant privacy or use of data from a third party—those must be specified.

Reviewer #1: Yes

Reviewer #2: Yes

**********

5. Is the manuscript presented in an intelligible fashion and written in standard English?

PLOS ONE does not copyedit accepted manuscripts, so the language in submitted articles must be clear, correct, and unambiguous. Any typographical or grammatical errors should be corrected at revision, so please note any specific errors here.

Reviewer #1: Yes

Reviewer #2: Yes

**********

6. Review Comments to the Author

Please use the space provided to explain your answers to the questions above. You may also include additional comments for the author, including concerns about dual publication, research ethics, or publication ethics. (Please upload your review as an attachment if it exceeds 20,000 characters)

Reviewer #1: Dear Editor,

Thank you for the opportunity to review this paper once more. I commend the authors in their efforts in addressing the previous comments and making the paper publication-ready. How ever, i have a few minor comments.

Minor comments:

Line 88, It would be better to say significantly reduce adverse birth outcomes as logically, it is not possible to halt adverse birth outcomes and their consequences in a country.

line 173, preterm pregnancy, i would suggest consistency in the use of terms throughout the paper, other sections of the paper use preterm birth.

It would be worthwhile stating the discrepancy in your findings regarding the association between twin pregnancies, low birth weight and preterm delivery together. While some sections of your discussion mention them together as possible risk factors for adverse outcomes, your findings seem to contradict this.

Reviewer #2: Authors adequately addressed prior concerns; methods and interpretation now sound. Recommend acceptance; only minor language/typo edits remain.

**********

7. PLOS authors have the option to publish the peer review history of their article (what does this mean?). If published, this will include your full peer review and any attached files.

If you choose “no”, your identity will remain anonymous but your review may still be made public.

**Do you want your identity to be public for this peer review?**   For information about this choice, including consent withdrawal, please see our Privacy Policy.

Reviewer #1: **Yes:**   Aliyou Moustapha Chandini

Reviewer #2: No

**********

[NOTE: If reviewer comments were submitted as an attachment file, they will be attached to this email and accessible via the submission site. Please log into your account, locate the manuscript record, and check for the action link "View Attachments". If this link does not appear, there are no attachment files.]

To ensure your figures meet our technical requirements, please review our figure guidelines: https://journals.plos.org/plosone/s/figures

You may also use PLOS’s free figure tool, NAAS, to help you prepare publication quality figures: https://journals.plos.org/plosone/s/figures#loc-tools-for-figure-preparation.

NAAS will assess whether your figures meet our technical requirements by comparing each figure against our figure specifications.

# Author comment: Author response to Decision Letter 2

**DOI:** 10.1371/journal.pone.0351676.r004

13 Apr 2026

Reviewer’s Comments

Reviewer #1

Comment: Line 88, It would be better to say significantly reduce adverse birth outcomes, as logically, it is not possible to halt adverse birth outcomes and their consequences in a country.

Response: Thank you for your comment. We have changed halt to significantly reduce based on your advice in the revised manuscript (line 88).

Comment: Line 173, preterm pregnancy, I would suggest consistency in the use of terms throughout the paper, other sections of the paper use preterm birth.

Response: Thank you for your valuable comment. We have corrected “preterm pregnancy” to “preterm birth” (line 173). Additionally, “preterm labor” has been revised to “preterm birth” in line 380 of the revised manuscript. We have carefully reviewed the entire document to ensure consistency, and no discrepancies remain; the terms “preterm birth” and “preterm delivery” are now used appropriately according to the context.

Comment: It would be worthwhile stating the discrepancy in your findings regarding the association between twin pregnancies, low birth weight and preterm delivery together. While some sections of your discussion mention them together as possible risk factors for adverse outcomes, your findings seem to contradict this.

Response: We thank the reviewer for this valuable comment and for highlighting the need for clearer interpretation. We acknowledge the apparent inconsistency between the established role of twin pregnancies as risk factors for adverse outcomes and the inverse association observed with preterm birth in our findings.

In response, we have revised the preterm birth section of the Discussion (lines 345–348) to explicitly address this discrepancy and to clarify that the observed inverse association contrasts with existing evidence and should be interpreted with caution.

Additionally, we have revised the wording in the adverse birth outcome section by explicitly referring to the outcome as a composite adverse birth outcome (line 439). This clarification ensures that the interpretation reflects the combined nature of the indicator, rather than implying a uniform effect across all individual outcomes, thereby reducing potential confusion for readers.

Reviewer #2

Comment: Authors adequately addressed prior concerns; methods and interpretation now sound. Recommend acceptance; only minor language/typo edits remain.

Response: Thank you for the comment. The manuscript has been revised for the typo and other language correction.

Attachment Submitted filename: Response to Reviewers.docx

# Editor report: Decision Letter 2

**DOI:** 10.1371/journal.pone.0351676.r005

**Authors:** Md. Obaidur Rahman

6 May 2026

PONE-D-25-43758R2

Prevalence and risk factors of adverse birth outcomes in Bangladesh: Insight from a nationwide survey

PLOS One

Dear Dr. Islam,

Thank you for submitting your manuscript to PLOS ONE. After careful consideration, we feel that it has merit but does not fully meet PLOS ONE’s publication criteria as it currently stands. Therefore, we invite you to submit a revised version of the manuscript that addresses the points raised during the review process.

Please submit your revised manuscript by Jun 20 2026 11:59PM. If you will need more time than this to complete your revisions, please reply to this message or contact the journal office at plosone@plos.org. When you're ready to submit your revision, log on to https://www.editorialmanager.com/pone/ and select the 'Submissions Needing Revision' folder to locate your manuscript file.

Please include the following items when submitting your revised manuscript:

- A letter that responds to each point raised by the academic editor and reviewer(s). You should upload this letter as a separate file labeled 'Response to Reviewers'.
- A marked-up copy of your manuscript that highlights changes made to the original version. You should upload this as a separate file labeled 'Revised Manuscript with Track Changes'.
- An unmarked version of your revised paper without tracked changes. You should upload this as a separate file labeled 'Manuscript'.

If you would like to make changes to your financial disclosure, please include your updated statement in your cover letter. Guidelines for resubmitting your figure files are available below the reviewer comments at the end of this letter.

If applicable, we recommend that you deposit your laboratory protocols in protocols.io to enhance the reproducibility of your results. Protocols.io assigns your protocol its own identifier (DOI) so that it can be cited independently in the future. For instructions see: https://journals.plos.org/plosone/s/submission-guidelines#loc-laboratory-protocols. Additionally, PLOS ONE offers an option for publishing peer-reviewed Lab Protocol articles, which describe protocols hosted on protocols.io. Read more information on sharing protocols at https://plos.org/protocols?utm_medium=editorial-email&utm_source=authorletters&utm_campaign=protocols.

As the corresponding author, your ORCID iD is verified in the submission system and will appear in the published article. PLOS supports the use of ORCID, and we encourage all coauthors to register for an ORCID iD and use it as well. Please encourage your coauthors to verify their ORCID iD within the submission system before final acceptance, as unverified ORCID iDs will not appear in the published article. *Only*   the individual author can complete the verification step; PLOS staff *cannot*   verify ORCID iDs on behalf of authors.

We look forward to receiving your revised manuscript.

Kind regards,

Md. Obaidur Rahman, Ph.D.

Academic Editor

PLOS One

**Journal Requirements:**

1. If the reviewer comments include a recommendation to cite specific previously published works, please review and evaluate these publications to determine whether they are relevant and should be cited. There is no requirement to cite these works unless the editor has indicated otherwise.

2. Please review your reference list to ensure that it is complete and correct. If you have cited papers that have been retracted, please include the rationale for doing so in the manuscript text, or remove these references and replace them with relevant current references. Any changes to the reference list should be mentioned in the rebuttal letter that accompanies your revised manuscript. If you need to cite a retracted article, indicate the article’s retracted status in the References list and also include a citation and full reference for the retraction notice.

**Additional Editor Comments:**

The authors have addressed many of the reviewer comments; however, some issues remain unresolved. In particular, discrepancies persist in the use of terminology related to “preterm birth” between the main text and figures. Please ensure consistent usage throughout the manuscript.

In addition, please standardize the capitalization format (sentence case vs. title case) across all figure and table titles in accordance with the journal’s guidelines.

[Note: HTML markup is below. Please do not edit.]

[NOTE: If reviewer comments were submitted as an attachment file, they will be attached to this email and accessible via the submission site. Please log into your account, locate the manuscript record, and check for the action link "View Attachments". If this link does not appear, there are no attachment files.]

To ensure your figures meet our technical requirements, please review our figure guidelines: https://journals.plos.org/plosone/s/figures

You may also use PLOS’s free figure tool, NAAS, to help you prepare publication quality figures: https://journals.plos.org/plosone/s/figures#loc-tools-for-figure-preparation.

NAAS will assess whether your figures meet our technical requirements by comparing each figure against our figure specifications.

# Author comment: Author response to Decision Letter 3

**DOI:** 10.1371/journal.pone.0351676.r006

18 May 2026

Response to Journal Requirements:

1. If the reviewer comments include a recommendation to cite specific previously published works, please review and evaluate these publications to determine whether they are relevant and should be cited. There is no requirement to cite these works unless the editor has indicated otherwise.

Response: We thank the editor for this note. We carefully reviewed all reviewer comments and confirm that no recommendations were made to cite specific previously published works.

2. Please review your reference list to ensure that it is complete and correct. If you have cited papers that have been retracted, please include the rationale for doing so in the manuscript text, or remove these references and replace them with relevant current references. Any changes to the reference list should be mentioned in the rebuttal letter that accompanies your revised manuscript. If you need to cite a retracted article, indicate the article’s retracted status in the References list and also include a citation and full reference for the retraction notice.

Response: We carefully reviewed the entire reference list to ensure that all references are complete, accurate, and up to date. We also checked for any retracted publications and confirm that no retracted articles have been cited in the manuscript.

Response to Additional Editor Comments:

1. The authors have addressed many of the reviewer comments; however, some issues remain unresolved. In particular, discrepancies persist in the use of terminology related to “preterm birth” between the main text and figures. Please ensure consistent usage throughout the manuscript.

Response: Thank you for this important observation. We have carefully revised the manuscript to ensure consistent use of the term “preterm birth” throughout the text and figures. In addition, the term “preterm delivery” was replaced with “preterm birth” to maintain terminological consistency across the manuscript.

2. In addition, please standardize the capitalization format (sentence case vs. title case) across all figure and table titles in accordance with the journal’s guidelines.

Response: Thank you for this valuable suggestion. We have carefully revised all figure and table titles throughout the manuscript and standardized their capitalization format in accordance with the journal’s guidelines. Specifically, sentence case formatting has now been applied across all figure and table titles.

Attachment Submitted filename: Response to Journal Requirements_R3.docx

# Editor report: Decision Letter 3

**DOI:** 10.1371/journal.pone.0351676.r007

**Authors:** Md. Obaidur Rahman

31 May 2026

Prevalence and risk factors of adverse birth outcomes in Bangladesh: Insight from a nationwide survey

PONE-D-25-43758R3

Dear Dr. Islam,

We’re pleased to inform you that your manuscript has been judged scientifically suitable for publication and will be formally accepted for publication once it meets all outstanding technical requirements.

Within one week, you’ll receive an e-mail detailing the required amendments. When these have been addressed, you’ll receive a formal acceptance letter and your manuscript will be scheduled for publication.

An invoice will be generated when your article is formally accepted. Please note, if your institution has a publishing partnership with PLOS and your article meets the relevant criteria, all or part of your publication costs will be covered. Please make sure your user information is up-to-date by logging into Editorial Manager at Editorial Manager® and clicking the ‘Update My Information' link at the top of the page. For questions related to billing, please contact billing support.

If your institution or institutions have a press office, please notify them about your upcoming paper to help maximize its impact. If they’ll be preparing press materials, please inform our press team as soon as possible -- no later than 48 hours after receiving the formal acceptance. Your manuscript will remain under strict press embargo until 2 pm Eastern Time on the date of publication. For more information, please contact onepress@plos.org.

Kind regards,

Md. Obaidur Rahman, Ph.D.

Academic Editor

PLOS One

Additional Editor Comments (optional):

Reviewers' comments:

# Editor report: Acceptance letter

**DOI:** 10.1371/journal.pone.0351676.r008

**Authors:** Md. Obaidur Rahman

PONE-D-25-43758R3

PLOS One

Dear Dr. Islam,

I'm pleased to inform you that your manuscript has been deemed suitable for publication in PLOS One. Congratulations! Your manuscript is now being handed over to our production team.

At this stage, our production department will prepare your paper for publication. This includes ensuring the following:

* All references, tables, and figures are properly cited

* All relevant supporting information is included in the manuscript submission,

* There are no issues that prevent the paper from being properly typeset

You will receive further instructions from the production team, including instructions on how to review your proof when it is ready. Please keep in mind that we are working through a large volume of accepted articles, so please give us a few days to review your paper and let you know the next and final steps.

Lastly, if your institution or institutions have a press office, please let them know about your upcoming paper now to help maximize its impact. If they'll be preparing press materials, please inform our press team within the next 48 hours. Your manuscript will remain under strict press embargo until 2 pm Eastern Time on the date of publication. For more information, please contact onepress@plos.org.

You will receive an invoice from PLOS for your publication fee after your manuscript has reached the completed accept phase. If you receive an email requesting payment before acceptance or for any other service, this may be a phishing scheme. Learn how to identify phishing emails and protect your accounts at https://explore.plos.org/phishing.

If we can help with anything else, please email us at customercare@plos.org.

Thank you for submitting your work to PLOS ONE and supporting open access.

Kind regards,

PLOS ONE Editorial Office Staff

on behalf of

Dr. Md. Obaidur Rahman

Academic Editor

PLOS One
